# Ginsenoside Rb2 promotes p62-mediated LDHA autophagic degradation to inhibit H3K18la-dependent PD-L1 transcription in cervical cancer

**DOI:** 10.3389/fonc.2026.1962784

**Published:** 2026-09-28

**Authors:** Xingwei Wang, Ziyue Zhang, Mei Chen, Liu Luo, Ru Sun, Chen Chen, Minmin Yu

**Affiliations:** 1 Department of Gynecology, The Second Hospital of Nanjing, Affiliated to Nanjing University of Chinese Medicine, Nanjing, China; 2 Department of Gynecology, Nanjing University of Chinese Medicine, Nanjing, China; 3 Department of Obstetrics and Gynecology, The Affiliated Hospital of Yangzhou University, Yangzhou, China; 4 Department of Gynecology, Nanjing Women and Children’s Healthcare Hospital, Women’s Hospital of Nanjing Medical University, Nanjing, Jiangsu, China; 5 School of Traditional Chinese Medicine, Faculty of Medicine, Yangzhou University, Yangzhou, China; 6 Jiangsu Cancer Hospital, The Affiliated Cancer Hospital of Nanjing Medical University, Nanjing, Jiangsu, China

**Keywords:** autophagic degradation, cervical cancer, ginsenoside Rb2, H3K18la, PD-L1

## Abstract

**Background:**

Ginsenoside Rb2 has demonstrated antitumor activity in multiple malignancies, yet its role in cervical cancer and the underlying mechanism remain unclear. This study investigated the effects of Rb2 on cervical cancer progression and explored whether metabolic reprogramming contributes to PD-L1 regulation.

**Methods:**

Cell proliferation, migration, invasion, glycolytic activity, lactate production, histone lactylation, and PD-L1 expression were evaluated in cervical cancer cells using CCK-8, EdU, colony formation, wound-healing, Transwell, Seahorse analysis, RT-qPCR, western blotting, flow cytometry, and ChIP-qPCR. LDHA regulation was examined by protein and mRNA stability assays, pharmacological inhibition, lysosome immunoprecipitation, gain- and loss-of-function studies, co-immunoprecipitation, molecular docking, and biotinylated Rb2 pull-down assays. T-cell function was assessed by Jurkat T-cell–tumor cell co-culture and IL-2 ELISA. Xenograft models and clinical samples were used for validation.

**Results:**

Rb2 significantly inhibited cervical cancer cell proliferation, migration, invasion, and xenograft tumor growth, reducing HeLa and SiHa cell viability by approximately 23.4% and 25.7%, respectively. Rb2 selectively downregulated PD-L1 and suppressed glycolysis and lactate production, with reduced H3K18la/H4K12la lactylation at the PD-L1 promoter. LDHA was the primary glycolytic enzyme reduced by Rb2, without changes in its mRNA. Rb2 decreased LDHA protein stability and promoted its autophagy-lysosome-dependent degradation, with p62 required and the p62–LDHA interaction enhanced by Rb2. Rb2-pretreated tumor cells restored IL-2 production by co-cultured Jurkat T cells, whereas LDHA overexpression reversed this effect. LDHA overexpression partially rescued Rb2’s effects, and LDHA correlated positively with PD-L1 in cervical cancer tissues.

**Conclusions:**

Rb2 suppresses cervical cancer progression by promoting p62-dependent autophagic degradation of LDHA, thereby inhibiting glycolysis-associated H3K18la and PD-L1 transcription. These findings reveal a p62/LDHA/H3K18la/PD-L1 signaling axis through which Rb2 exerts its antitumor effects.

## Introduction

Cervical cancer remains one of the most common gynecological malignancies and a leading cause of cancer-related death among women worldwide, particularly in developing countries (1). Although advances in human papillomavirus vaccination, screening programs, radical surgery, radiotherapy, and platinum-based chemotherapy have improved disease management, patients with recurrent, metastatic, or treatment-refractory cervical cancer still face limited therapeutic options and poor clinical outcomes (2, 3). Beyond survival outcomes, the psychological burden associated with multimodal treatment of patients with gynecological cancer further underscores the need for patient-centered management strategies (4). Increasing evidence indicates that, beyond uncontrolled proliferation and metastasis, immune evasion is a critical hallmark of cervical cancer progression (5, 6). Among immune checkpoint molecules, programmed death-ligand 1 (PD-L1) has emerged as a central suppressor of antitumor immunity by inhibiting T-cell activation and promoting an immunosuppressive tumor microenvironment (7). Elevated PD-L1 expression has been associated with disease progression, treatment resistance, and unfavorable prognosis in cervical cancer, highlighting the need to elucidate the mechanisms governing its dysregulation (8).

Immune checkpoint blockade has revolutionized cancer therapy by restoring exhausted T-cell function and reinvigorating antitumor immune responses, with the PD-1/PD-L1 axis representing one of the most successful therapeutic targets (9). In cervical cancer, immunotherapy has shown considerable promise, particularly in patients with persistent, recurrent, or metastatic disease, and PD-1/PD-L1 inhibitors have been incorporated into clinical treatment paradigms (10). However, the overall response rate remains modest, and only a subset of patients derives durable benefit. Intratumoral heterogeneity, adaptive immune resistance, compensatory immunosuppressive pathways, and treatment-related adverse effects all limit the efficacy of current checkpoint inhibitors (11). These challenges suggest that direct antibody-mediated blockade alone may be insufficient and that identifying upstream molecular determinants of PD-L1 expression may provide a more effective strategy to improve therapeutic responsiveness and overcome immune escape in cervical cancer.

Epigenetic reprogramming has emerged as a fundamental mechanism linking oncogenic signaling, metabolic remodeling, and immune regulation in cancer (12). In addition to canonical DNA methylation and histone acetylation or methylation, histone lactylation has recently been recognized as a novel metabolic-epigenetic modification that directly couples lactate accumulation to transcriptional activation (13). Because cervical cancer cells exhibit prominent glycolytic reprogramming and increased lactate production, histone lactylation may serve as a critical molecular bridge between metabolic dysfunction and immune checkpoint activation (14). Indeed, accumulating studies have established that epigenetic dysregulation contributes extensively to cervical carcinogenesis, tumor progression, therapy resistance, and immune remodeling (15–17). However, whether histone lactylation directly regulates PD-L1 transcription in cervical cancer remains unclear. More importantly, the specific histone lactylation marks involved and their upstream metabolic drivers have not yet been defined. This unresolved question represents a critical knowledge gap in understanding how metabolic reprogramming shapes immune escape in cervical cancer.

Lactate dehydrogenase A (LDHA) is a key glycolytic enzyme that catalyzes the conversion of pyruvate into lactate and is therefore a major determinant of intracellular lactate availability and lactylation signaling (18). Aberrant LDHA expression has been implicated in multiple malignant phenotypes, including enhanced proliferation, invasion, metastasis, metabolic adaptation, and immune suppression (19). In cervical cancer, elevated LDHA expression has been associated with aggressive biological behavior and poor clinical outcome, underscoring its potential as a therapeutic target (20). Beyond transcriptional regulation, LDHA can be controlled at the post-translational level through multiple mechanisms, including ubiquitination and protein degradation (21). Selective autophagy, particularly p62/SQSTM1-mediated cargo recognition and lysosomal delivery, has emerged as an important pathway for regulating the turnover of oncogenic and metabolic proteins (22). Nevertheless, whether LDHA is subject to p62-dependent autophagic degradation in cervical cancer, and whether such regulation impacts histone lactylation-driven PD-L1 expression, has not been elucidated. Moreover, although several LDHA-targeting compounds have shown preclinical activity, their translational potential is often hindered by limited specificity, metabolic compensation, and safety concerns, highlighting the need for alternative approaches to modulate LDHA-centered metabolic signaling (23).

Natural products represent an important source of bioactive compounds for cancer therapy because of their structural diversity, multitarget activities, and relatively favorable safety profiles (24, 25). Increasing evidence suggests that natural small molecules can modulate metabolic pathways and epigenetic states, thereby reshaping oncogenic transcriptional programs and tumor immune phenotypes (26, 27). Ginsenoside Rb2, a major pharmacologically active saponin derived from Panax ginseng, has been reported to exert antitumor and metabolism-regulatory effects in several malignancies (28, 29); however, its role in cervical cancer remains incompletely characterized. More importantly, whether Rb2 can target the metabolic-epigenetic regulation of PD-L1 has not been investigated. In the present study, we demonstrate that Rb2 suppresses cervical cancer progression through a p62/LDHA/H3K18la/PD-L1 signaling axis. Our findings provide mechanistic insight into the metabolic-epigenetic control of immune checkpoint expression and support the therapeutic potential of Rb2 in cervical cancer.

## Materials and methods

### Cell lines and cell culture

Human cervical cancer cell lines (CaSki, HeLa, SiHa, and C33A) and normal human cervical epithelial cells (HcerEpic) were purchased from the American Type Culture Collection (ATCC), and the mouse cervical cancer cell line U14 was obtained from the Cell Bank of the Chinese Academy of Sciences. All human cell lines were authenticated by short tandem repeat (STR) profiling, while the murine U14 cell line was verified by species identification. All cell lines were routinely tested and found negative for mycoplasma contamination. Cells were cultured in DMEM or RPMI-1640 medium supplemented with 10% fetal bovine serum and penicillin/streptomycin, at 37 °C in a humidified atmosphere containing 5% CO2.

### Human cervical cancer tissue samples

Cervical cancer tissue samples used in this study were collected from January 2020 to December 2024 from the Gynecological Specimen Bank of the Affiliated Hospital of Yangzhou University. The study protocol was approved by the Ethics Committee of the Affiliated Hospital of Yangzhou University (Approval No. 2025-YKL08-K04). Written informed consent was obtained from all patients. Tumor tissues were collected immediately after surgical resection, snap-frozen in liquid nitrogen, and stored at −80 °C until further use.

### Cell counting kit-8 assay

Cell viability was evaluated using a CCK-8 kit (Abbkine, Wuhan, China). HcerEpic, CaSki, HeLa, SiHa, and C33A cells were seeded into 96-well plates at a density of 3 × 10^3^ cells per well and treated with Rb2 (HY-N0040, MedChemExpress, Monmouth Junction, NJ, USA) at final concentrations of 0, 2, 5, or 10 μM. After 48 h of incubation at 37 °C in 5% CO_2_, 10 μL of CCK-8 reagent was added to each well, followed by an additional 2 h incubation. Absorbance at 450 nm was measured using a multimode microplate reader (Thermo Fisher Scientific, USA).

### 5-ethynyl-2′-deoxyuridine proliferation assay

Cell proliferation was assessed using an EdU assay. Cells were seeded into 96-well plates at a density of 3 × 10^3^ cells per well and treated with 5 μM Rb2 for 48 h. The culture medium was then replaced with fresh medium containing 10 μM EdU (Abbkine, Wuhan, China), and the cells were incubated for another 2 h. After removal of the medium, cells were fixed with 4% paraformaldehyde and permeabilized with 0.5% Triton X-100 in PBS for 10 min. Cells were then incubated with Click reaction solution in the dark for 30 min, followed by nuclear counterstaining with DAPI. Images were captured using an Olympus optical microscope (Tokyo, Japan). DAPI-stained nuclei appeared blue, and proliferating EdU-positive cells displayed red fluorescence. The proliferation rate was determined by calculating the percentage of EdU-positive cells. Quantitative analysis of total and proliferating cells was performed using ImageJ software.

### Colony formation assay

Colony formation capacity was used to evaluate long-term proliferative ability. Cells were seeded into 6-well plates at a density of 400 cells per well and cultured in complete medium for 14 days. After visible colonies had formed, cells were fixed with 4% paraformaldehyde and stained with crystal violet. Colony numbers were quantified using ImageJ software.

### Wound healing assay

Cells were seeded into 6-well plates and cultured until reaching 90% confluence. A linear wound was created in the cell monolayer using a sterile 200 μL pipette tip. Detached cells were gently removed by washing with PBS, and serum-free medium was then added. Images were captured at 0 and 48 h under an inverted microscope. Wound width was measured using ImageJ software, and the migration rate was calculated accordingly.

### Transwell assay

Cells were resuspended in serum-free medium and seeded into the upper chamber of Transwell inserts (LABSELECT, China), while medium containing 10% FBS was added to the lower chamber as a chemoattractant. After 48 h of incubation, the inserts were removed, and cells were fixed with 4% paraformaldehyde and stained with 0.1% crystal violet. Non-migrated/non-invaded cells on the upper surface were gently removed with a cotton swab. Cells on the lower surface were observed and counted in randomly selected microscopic fields under an inverted microscope. Quantification was performed using ImageJ software.

### Flow cytometry

Flow cytometry was used to quantify cell-surface PD-L1 expression. Log-phase cells were collected, washed with PBS, and resuspended to a density of 1 × 10^6^ cells/mL. A total of 100 μL of cell suspension was incubated with FITC anti-human PD-L1 antibody (393605, BioLegend, USA) at 4 °C for 30 min in the dark. Isotype control and unstained control groups were included in parallel. After incubation, cells were washed twice with pre-chilled PBS to remove unbound antibody and finally resuspended in 300 μL PBS. Samples were analyzed using a BD FACSVerse™ flow cytometer, and fluorescence intensity was quantified using FlowJo software. PD-L1 expression was presented as mean fluorescence intensity (MFI).

### T cell-tumor cell co-culture assay

For T-cell functional assays, Jurkat T cells (clone E6-1, ATCC) were co-cultured with Rb2-pretreated or control HeLa/SiHa cells at a tumor-to-T-cell ratio of 1:10 for 24 h. Tumor cells were pretreated with 5 μM Rb2 for 48 h and then washed three times with PBS to exclude any direct Rb2 carryover effect on T cells; the final wash supernatant was collected as a washout control. Where indicated, a PD-L1 neutralizing antibody (BioLegend, USA) was added at 5 μg/mL during co-culture. After co-culture, culture supernatants were collected, and IL-2 levels were quantified using an ELISA kit according to the manufacturer’s instructions.

### Cellular energy metabolism analysis

Extracellular acidification rate (ECAR) was measured using the Seahorse XF24 Extracellular Flux Analyzer (Agilent, USA). Briefly, 1 × 10^5^ cells were resuspended in 250 μL complete medium and seeded into Seahorse XF24 cell culture microplates, followed by overnight incubation at 37 °C. Before the assay, the medium was replaced with XF assay medium containing 2 mM glutamine. Glucose (30 mM), oligomycin (2 μM), and 2-deoxy-D-glucose (2-DG, 100 mM) were sequentially injected, and dynamic changes in ECAR were recorded in real time. All data were normalized to cell number before statistical analysis.

### Lactate measurement

Lactate concentration was quantified using a lactate assay kit (Abbkine, Wuhan, China). Briefly, lactate in the samples reacted enzymatically with the assay reagents to generate a specific chromogenic product. After the addition of detection probe, absorbance was measured at 570 nm using a microplate reader (Thermo Fisher, USA), and lactate concentration was calculated according to the standard curve.

### Quantitative reverse transcription PCR

Total RNA was extracted from cells or tissues using RNA-Easy Isolation Reagent (Vazyme, Nanjing, China). Equal amounts of RNA were reverse-transcribed into cDNA using HiScript II Q RT SuperMix for qPCR (+gDNA Wiper) (Vazyme, Nanjing, China). Quantitative PCR was performed using ChamQ Universal SYBR qPCR Master Mix (Vazyme, Nanjing, China) with gene-specific primers on a real-time PCR system according to the manufacturer’s instructions. Relative mRNA expression levels were calculated using the 2^^−ΔΔCt^ method, with β-actin as the internal control. Primer sequences were as follows: PD-L1 forward:5’- TGGCATTTGCTGAACGCATTT -3’; PD-L1 reverse:5’- TGCAGCCAGGTCTAATTGTTTT -3’; CD24 forward:5’- CTCCTACCCACGCAGATTTATTC-3’; CD24 reverse:5’- AGAGTGAGACCACGAAGAGAC-3’; CD47 forward:5’- AGAAGGTGAAACGATCATCGAGC-3’; CD47 reverse:5’- CTCATCCATACCACCGGATCT-3’; CD155 forward:5’- TGGAGGTGACGCATGTGTC-3’; CD155 reverse:5’- GTTTGGACTCCGAATAGCTGG-3’; PSGL1 forward:5’- TGTTGCTGATCCTACTGGGC-3’; PSGL1 reverse:5’- CACAGTGGTAGACTCAGGGGT -3’; CEACAM1 forward:5’- TGCTCTGATAGCAGTAGCCCT-3’; CEACAM1 reverse:5’- TGCCGGTCTTCCCGAAATG-3’; HK1 forward:5’-GCTCTCCGATGAAACTCTCATAG-3’; HK1 reverse:5’-GGACCTTACGAATGTTGGCAA-3’; HK2 forward:5’-GAGCCACCACTCACCCTACT-3’; HK2 reverse:5’-CCAGGCATTCGGCAATGTG-3’; PFKL forward:5’- GCTGGGCGGCACTATCATT-3’; PFKL reverse:5’- TCAGGTGCGAGTAGGTCCG-3’; PFKM forward:5’- GGTGCCCGTGTCTTCTTTGT-3’; PFKM reverse:5’- AAGCATCATCGAAACGCTCTC-3’; PFKP forward:5’- GCATGGGTATCTACGTGGGG-3’; PFKP reverse:5’- CTCTGCGATGTTTGAGCCTC-3’; PKM1 forward:5’- CAGCCAAAGGGGACTATCCT-3’; PKM1 reverse:5’- GAGGCTCGCACAAGTTCTTC-3’; PKM2 forward:5’- CTATCCTCTGGAGGCTGTGC-3’; PKM2 reverse:5’- GTGGGGTCGCTGGTAATG-3’; LDHA forward:5’- ATGGCAACTCTAAAGGATCAGC-3’; LDHA reverse:5’- CCAACCCCAACAACTGTAATCT-3’; LDHB forward:5’- TGGTATGGCGTGTGCTATCAG-3’; LDHB reverse:5’- TTGGCGGTCACAGAATAATCTTT-3’; β-actin forward:5’-CTCCATCCTGGCCTCGCTGT-3’; β-actin reverse:5’-GCTGTCACCTTCACCGTTCC-3’.

### Western blotting

Cells were lysed in RIPA buffer (Servicebio, Wuhan, China) supplemented with protease inhibitors (Beyotime, Shanghai, China). Protein concentration was determined using a BCA protein assay kit (Thermo Fisher Scientific, USA). Equal amounts of protein were separated by SDS-PAGE and transferred onto PVDF membranes (Millipore, USA). Membranes were incubated with primary antibodies overnight at 4 °C, followed by incubation with HRP-conjugated secondary antibodies at room temperature. Protein bands were visualized using ECL chemiluminescence reagents (Abbkine, Wuhan, China) and imaged with a ChemiDoc imaging system (Bio-Rad, USA). β-actin was used as the loading control, and band intensity was quantified by densitometric analysis.

The primary antibodies used were as follows: PD-L1 (28076-1-AP, Proteintech, Wuhan, China), pan-Kla (PTM-1401RM, PTM Bio, Hangzhou, China), H3K9la (PTM-1419RM, PTM Bio, Hangzhou, China), H3K18la (PTM-1406RM, PTM Bio, Hangzhou, China), H3K23la (PTM-1413RM, PTM Bio, Hangzhou, China), Histone H3 (PTM-1002RM, PTM Bio, Hangzhou, China), H4K5la (PTM-1407RM, PTM Bio, Hangzhou, China), H4K8la (PTM-1415RM, PTM Bio, Hangzhou, China), H4K12la (PTM-1411RM, PTM Bio, Hangzhou, China), H4K16la (PTM-1417RM, PTM Bio, Hangzhou, China), Histone H4 (PTM-1015RM, PTM Bio, Hangzhou, China), HK1 (#2024S, CST, USA), HK2 (#2867S, CST, USA), PFKL (#ER64567, HUABIO, Hangzhou, China), PFKM (#ET7106-97, HUABIO, Hangzhou, China), PFKP (#HA500472, HUABIO, Hangzhou, China), PKM1 (#7067S, CST, USA), PKM2 (#4053S, CST, USA), LDHA (#3582S, CST, USA), LDHB (#0807-1, HUABIO, Hangzhou, China), LAMP2 (66301-1-Ig, Proteintech, Wuhan, China), p62 (18420-1-AP, Proteintech, Wuhan, China), Ki67 (ab15580, Abcam, USA), and β-actin (AC026, ABclonal, Wuhan, China).

### Chromatin immunoprecipitation

Tumor cells were cross-linked with 1% formaldehyde in PBS at room temperature for 10 min, and cross-linking was quenched with 1.25 mol/L glycine. Cells were then washed and lysed on ice for 1 h in cold lysis buffer containing 1% SDS, 50 mmol/L Tris-HCl (pH 8.1), 5 mmol/L EDTA, and protease inhibitors. Chromatin was sonicated and centrifuged at 4 °C and 4000 rpm for 10 min, and the supernatant was collected. The chromatin solution was diluted with dilution buffer containing 1% Triton X-100, 20 mmol/L Tris-HCl (pH 8.1), 150 mmol/L NaCl, 2 mmol/L EDTA, and protease inhibitors. Chromatin samples were incubated overnight at 4 °C with 2 μg rabbit IgG or rabbit anti-H3K18la antibody. The following day, 30 μL Dynabeads™ Protein G magnetic beads (Invitrogen, USA) were added and incubated at 4 °C for an additional 4 h. After washing, the Input samples and immunoprecipitated complexes were de-crosslinked at 65 °C for 12 h. DNA was purified using the MinElute PCR Purification Kit (Qiagen, Germany). ChIP-qPCR was performed using PerfectStart Green qPCR SuperMix (TransGen Biotech, Beijing, China) on a QuantStudio 5 real-time PCR system. Primer sequences used for the PD-L1 promoter were: Forward: 5’-TGATCTGAAGGGCAGCATTTC-3’; Reverse: 5’-CTCCATCCCAAAGAAAGGG-3’; Primer sequences used for the c-Myc promoter were: Forward: 5’-TTCTGAAACCTGGCTGAGAAA-3’; Reverse: 5’-CTAGGGCGAGAGGGAGGTT-3’.

### Plasmid construction, RNA interference, and cell transfection

Overexpression plasmids, empty vectors, short hairpin RNAs (shRNAs), and wild-type plasmids used in this study were constructed by Nanjing Linghao Gene Biotechnology Co., Ltd. Briefly, the coding sequences of human LDHA and p62 were amplified by PCR and cloned into the pCDH-EF1a-MCS-CMV-T2A-Puro lentiviral overexpression vector, with Flag or His fusion tags. shRNA oligonucleotides were annealed and inserted into the LHSF: U6-shRNA-CBh-T2A-Puro vector. Recombinant plasmids together with packaging plasmids were co-transfected into HEK-293T cells using Polyplus transfection reagent (Sartorius, Germany). Lentiviral supernatants were collected 24–48 h after transfection, aliquoted, and stored at −80 °C. To establish stable cell lines, target cells were infected with lentivirus in the presence of 5 μg/mL polybrene (Beyotime, Shanghai, China) for 24 h, followed by puromycin selection to obtain positive clones. Knockdown or overexpression efficiency was confirmed by RT-qPCR or Western blotting.

### Protein and RNA stability assays

Cells from different treatment groups were seeded into 6-well plates at an appropriate density. When cell confluence reached approximately 70%, cells were treated with either cycloheximide (CHX, 100 μg/mL; HY-12320, MedChemExpress, Monmouth Junction, NJ, USA) or actinomycin D (ActD, 5 μg/mL; HY-17559, MedChemExpress, Monmouth Junction, NJ, USA). Cells were collected at 0, 3, 6, and 12 h after treatment. Protein and mRNA stability were evaluated by Western blotting and RT-qPCR, respectively.

### Detection of proteins degradation

Cells from different treatment groups were seeded into 6-well plates at an appropriate density. When cell confluence reached approximately 70%, cells were treated with 5 μM Rb2 in the presence or absence of MG-132 (10 μM; HY-13259, MedChemExpress, Monmouth Junction, NJ, USA) or chloroquine (50 μM; HY-17589A, MedChemExpress, Monmouth Junction, NJ, USA). After 48 h, cells were harvested, and target protein levels were analyzed by Western blotting.

### Lysosome immunoprecipitation

Cells were washed twice with pre-chilled PBS and collected in 1 mL PBS containing protease/phosphatase inhibitors. After centrifugation at 1000 ×g for 2 min at 4 °C, the cell pellet was resuspended in 800 μL PBS containing inhibitors, and 20 μL was reserved as the whole-cell control. The remaining suspension was mechanically homogenized using a 2 mL Dounce homogenizer. Homogenates were centrifuged at low speed (1000 ×g, 4 °C, 2 min) to remove cell debris and unbroken cells. The supernatant was then incubated with 10 μL anti-TMEM192 antibody and 100 μL Protein A/G magnetic beads for immunoprecipitation. After magnetic separation, immune complexes were washed three times with PBS and lysed in 100 μL ice-cold 1% Triton X-100 lysis buffer to extract bound proteins. The magnetic beads were then removed, and the target proteins were analyzed by Western blotting.

### Co-immunoprecipitation

To investigate protein-protein interactions, cells were pretreated with the proteasome inhibitor MG132 (10 μM) for 6 h before lysis. Cells were lysed with NP-40 lysis buffer (Beyotime, Shanghai, China) containing protease inhibitors, and the supernatants were collected after centrifugation. Protein expression was preliminarily examined by SDS-PAGE and Western blotting. Equal amounts of cell lysates were incubated with anti-LDHA antibody (4 μg/mL), anti-p62 antibody (4 μg/mL), or isotype control IgG (AC011, ABclonal, Wuhan, China), followed by incubation with Protein A/G magnetic beads (20241, Thermo Fisher, USA) for 3 h at 4 °C. After extensive washing with pre-chilled PBS, the immunoprecipitates were boiled in protein loading buffer and subjected to Western blotting with anti-LDHA and anti-p62 antibodies.

### Molecular docking

Molecular docking simulations were performed to assess the potential interactions of Rb2 with LDHA and p62. The three-dimensional crystal structures of LDHA and p62 were retrieved from the Protein Data Bank (PDB; LDHA PDB ID: 6ZZR; p62 PDB ID: 5YPG; https://www.rcsb.org), and the chemical structure of Rb2 was obtained from the PubChem database (https://pubchem.ncbi.nlm.nih.gov/). Docking analysis was conducted using AutoDock Vina1.2.5 (https://autodock.scripps.edu/). The predicted binding energy was calculated in kcal/mol, with lower values indicating more stable binding.

### Biotin labeling and pull-down assay

Rb2 was biotinylated by coupling its active group with activated biotin reagent. Briefly, Rb2 was reacted in acetonitrile with a carbodiimide coupling reagent and the catalyst 4-dimethylaminopyridine, followed by the addition of D-biotin. The reaction mixture was stirred under reflux for 4 h. The product was extracted with dichloromethane and purified by column chromatography using dichloromethane/methanol (20:1) as the eluent to obtain the white solid biotinylated Rb2 (Bio-Rb2). The resulting biotinylated small molecule was used for subsequent pull-down assays.

Bio-Rb2 or unlabeled Rb2 was incubated with streptavidin-coated magnetic beads (P2151, Beyotime, Shanghai, China) at room temperature for 25 min, followed by incubation with lysates from tumor cells with or without p62 overexpression for 6 h at 4 °C. Proteins bound to Bio-Rb2 were collected by magnetic separation, washed, and resuspended, and the interaction of Rb2 with LDHA and p62 was analyzed by Western blotting.

To validate the specificity of Bio-Rb2, competitive pull-down assays were performed by pre-incubating HeLa cell lysates with unlabeled free Rb2 at molar ratios of 1:5 and 1:10 (Bio-Rb2:free Rb2; corresponding to 10 μM Bio-Rb2 with 50 or 100 μM free Rb2) for 1 h at 4 °C before the addition of Bio-Rb2. The subsequent pull-down and Western blotting procedures were performed as described above.

### Subcutaneous xenograft model

All animal experiments were approved by the Animal Ethics Committee of Yangzhou University (No. 202507057). Six-week-old female BALB/c nude mice were used to establish subcutaneous xenograft models with human cervical cancer cell lines (HeLa or SiHa), while six-week-old female C57BL/6 mice were used for xenograft models with the murine cervical cancer cell line U14. Each mouse received a subcutaneous injection of 3 × 10^6^ cells. For the treatment experiment, C57BL/6 mice bearing U14 xenografts received drug administration, and they were divided into the following groups: a control group (intraperitoneal injection of PBS), an Rb2-treated group (intraperitoneal injection of Rb2 at 10 mg/kg once every 2 days), an OE-LDHA group (inoculated with LDHA-overexpressing cells and then treated with PBS), and an OE-LDHA+Rb2 group (inoculated with LDHA-overexpressing cells and then treated with Rb2 at the same regimen). After 30 days of treatment, all mice were sacrificed, and the xenograft tumors were excised, measured, weighed, and subjected to subsequent analyses. All mice were housed under specific pathogen-free (SPF) conditions. Body weight was recorded every 3 days. Tumor length and width were measured using calipers, and tumor volume was calculated using the formula: V = A × B^2^/2, where A represents the long diameter and B represents the short diameter.

Two complementary *in vivo* models were deliberately used for distinct experimental purposes: the nude mouse model with human HeLa/SiHa cells was designed to evaluate the intrinsic, cell-autonomous antitumor activity of Rb2 against human cervical cancer cells, whereas the immunocompetent C57BL/6 mouse model with syngeneic murine U14 cells was used for the *in vivo* rescue experiment to preserve an intact tumor immune microenvironment, which is required for evaluating PD-L1-dependent effects in a physiologically relevant setting. Mice were randomly assigned to experimental groups, and treatment administration and outcome measurements (tumor volume and weight) were performed by investigators blinded to group allocation. Mice were anesthetized with isoflurane (5% for induction, 1.5-2.5% for maintenance) for procedures requiring restraint and were euthanized by CO_2_ inhalation (20–30% chamber displacement rate per minute, followed by 5 min of exposure to confirm death, in accordance with the AVMA Guidelines for the Euthanasia of Animals) at the end of the experiments. All animal experiments were performed in accordance with the ARRIVE guidelines.

### Immunohistochemistry

Immunohistochemical staining was performed to detect the expression and localization of specific proteins in tumor tissues. Tumor samples were fixed in 4% paraformaldehyde, embedded in paraffin, and sectioned. After deparaffinization, rehydration, and PBS washing, sections were subjected to antigen retrieval. The sections were then incubated with specific primary antibodies overnight at 4 °C, followed by incubation with HRP-conjugated secondary antibodies at room temperature. Antigen-antibody complexes were visualized using a 3,3′-diaminobenzidine (DAB) detection system, and nuclei were counterstained with hematoxylin. Images were captured under a light microscope, and staining results were analyzed according to staining intensity and localization.

### Statistical analysis

Differences between two groups and among multiple groups were analyzed using unpaired two-tailed Student’s t-test and one-way or two-way analysis of variance (ANOVA), respectively. Normality was assessed using the Shapiro–Wilk test. For multiple-group comparisons, one-way ANOVA was followed by Tukey’s *post hoc* test, and two-way ANOVA was followed by Bonferroni *post hoc* test. Data are presented as mean ± standard deviation (SD). Statistical analyses were performed using GraphPad Prism 9.0 (GraphPad Software, San Diego, CA, USA). Bioinformatics analyses were conducted as described above. All *in vitro* experiments were performed with at least three independent biological replicates (n = 3), and *in vivo* experiments used n = 6 mice per group; the exact n values are stated in each figure legend. P value < 0.05 was considered statistically significant. Significance levels were defined as follows: *P < 0.05, **P < 0.01, and ns, not significant.

## Results

### Ginsenoside Rb2 suppresses cervical cancer cell proliferation, migration, and invasion *in vitro* and *in vivo*

To evaluate the effects of ginsenoside Rb2 on cervical cancer progression, four cervical cancer cell lines (CaSki, HeLa, SiHa, and C33A) and one normal human cervical epithelial cell line (HcerEpic) were treated with increasing concentrations of Rb2 (0, 2, 5, and 10 μM) for 24 or 48 h. CCK-8 assays showed that Rb2 reduced the viability of cervical cancer cells in a concentration- and time-dependent manner, whereas no significant cytotoxic effect was observed in HcerEpic cells under the same conditions (**Figure 1A**). To further determine whether Rb2 affects the proliferative capacity of cervical cancer cells, EdU incorporation and colony formation assays were performed. As shown in **Figures 1B, C**, Rb2 treatment markedly decreased the proportion of EdU-positive cells and reduced colony-forming ability, indicating a significant inhibitory effect on cervical cancer cell proliferation. We next examined whether Rb2 influences the migratory and invasive properties of cervical cancer cells. Wound-healing and Transwell assays consistently demonstrated that Rb2 significantly impaired the migration and invasion capacities of cervical cancer cells (**Figures 1D, E**). Given the inherent limitations of *in vitro* cell models, we further established a subcutaneous xenograft model in nude mice. *In vivo*, Rb2 treatment significantly reduced tumor volume and tumor weight, indicating a marked suppression of cervical cancer progression (**Figure 1F**). Taken together, these findings demonstrate that Rb2 exerts potent antitumor effects against cervical cancer both *in vitro* and *in vivo* by inhibiting cell proliferation, migration, invasion, and tumor growth.

**Figure 1:**
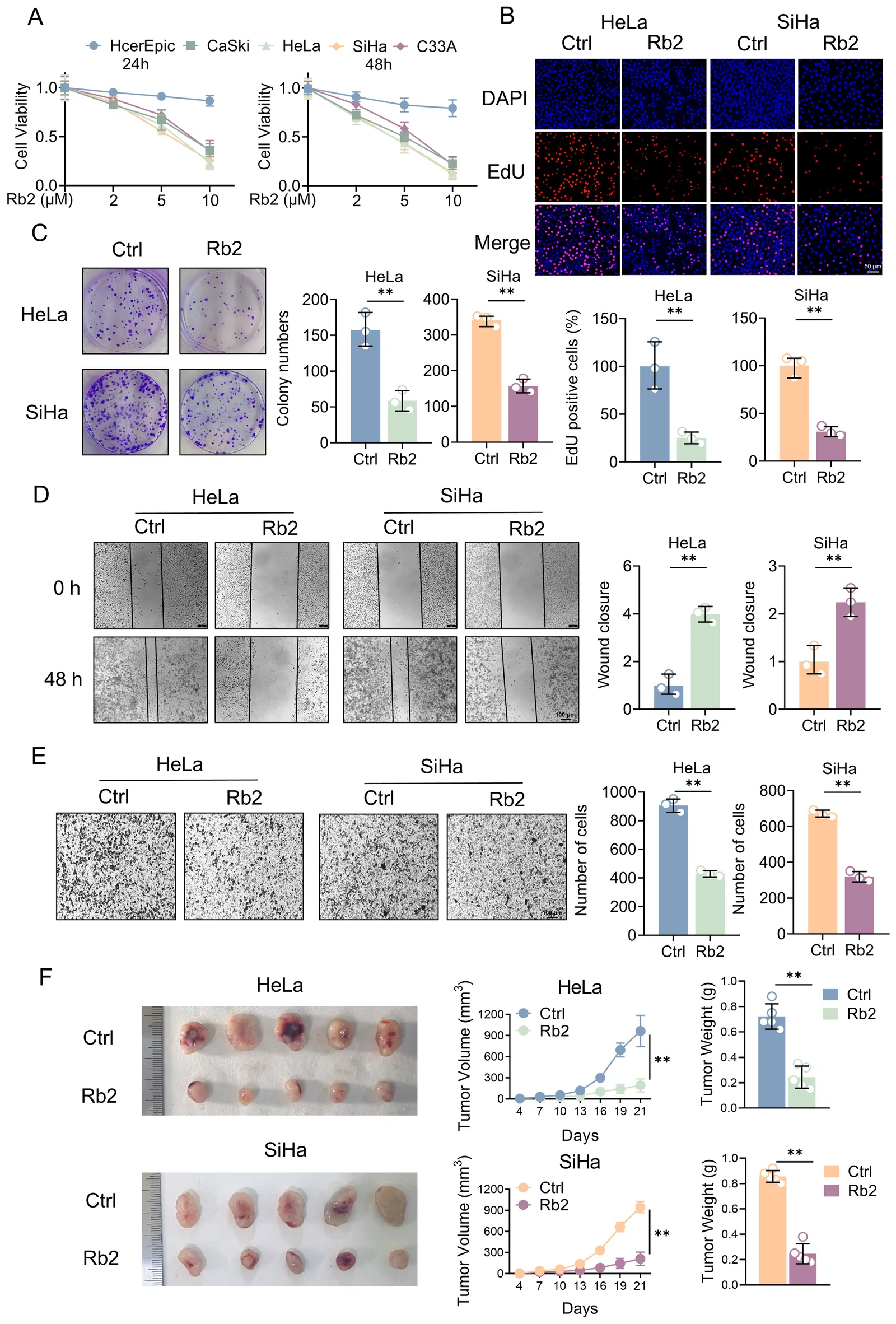
Ginsenoside Rb2 suppresses cervical cancer cell proliferation, migration, and invasion *in vitro* and *in vivo*. **(A)** Cell viability of normal cervical epithelial cells (HcerEpic) and cervical cancer cells (CaSki, HeLa, SiHa, and C33A) treated with the indicated concentrations of Rb2 for 24 h and 48 h, as assessed by CCK-8 assay. **(B)** EdU incorporation assay in HeLa and SiHa cells treated with Rb2. Representative fluorescence images of EdU (red), DAPI (blue), and merged signals are shown. Quantification of EdU-positive cells is presented on the right. **(C)** Colony formation assay in HeLa and SiHa cells treated with Rb2. Representative images and quantification of colony numbers are shown. **(D)** Wound-healing assay in HeLa and SiHa cells treated with Rb2 for 48 h. Representative images at 0 h and 48 h and quantification of wound closure are shown. **(E)** Transwell invasion assay in HeLa and SiHa cells treated with Rb2. Representative images of invaded cells (fixed and stained with 0.1% crystal violet) and corresponding quantification are shown. **(F)** Representative images of xenograft tumors derived from HeLa and SiHa cells in the control and Rb2-treated groups. Tumor growth curves and final tumor weights are shown on the right. Data are presented as mean ± SD from three independent biological replicates (n = 3) for *in vitro* assays, and n = 6 mice per group for the *in vivo* xenograft study. *P < 0.05, **P < 0.01.

### Ginsenoside Rb2 downregulates PD-L1 expression in cervical cancer cell

We next investigated whether the antitumor effect of Rb2 might be associated with modulation of the tumor microenvironment, particularly immune checkpoint molecules involved in immune evasion. To this end, the transcriptional levels of several representative immune checkpoints, including PD-L1 (CD274), CD24, CD47, CD155, PSGL1, and CEACAM1, were examined by RT-qPCR. Among these molecules, Rb2 treatment specifically and significantly reduced CD274 mRNA expression, whereas the other immune checkpoint genes were not substantially altered (**Figure 2A**). Western blot analysis further confirmed that Rb2 markedly decreased PD-L1 protein expression in cervical cancer cells (**Figure 2B**). To more directly assess cell-surface PD-L1 expression, flow cytometry was performed. Consistent with the above findings, Rb2 significantly reduced PD-L1 expression on the surface of cervical cancer cells (**Figure 2C**). Collectively, these results indicate that Rb2 selectively suppresses PD-L1 expression in cervical cancer cells, suggesting that attenuation of PD-L1-mediated immune escape may contribute to the inhibitory effect of Rb2 on cervical cancer progression.

**Figure 2:**
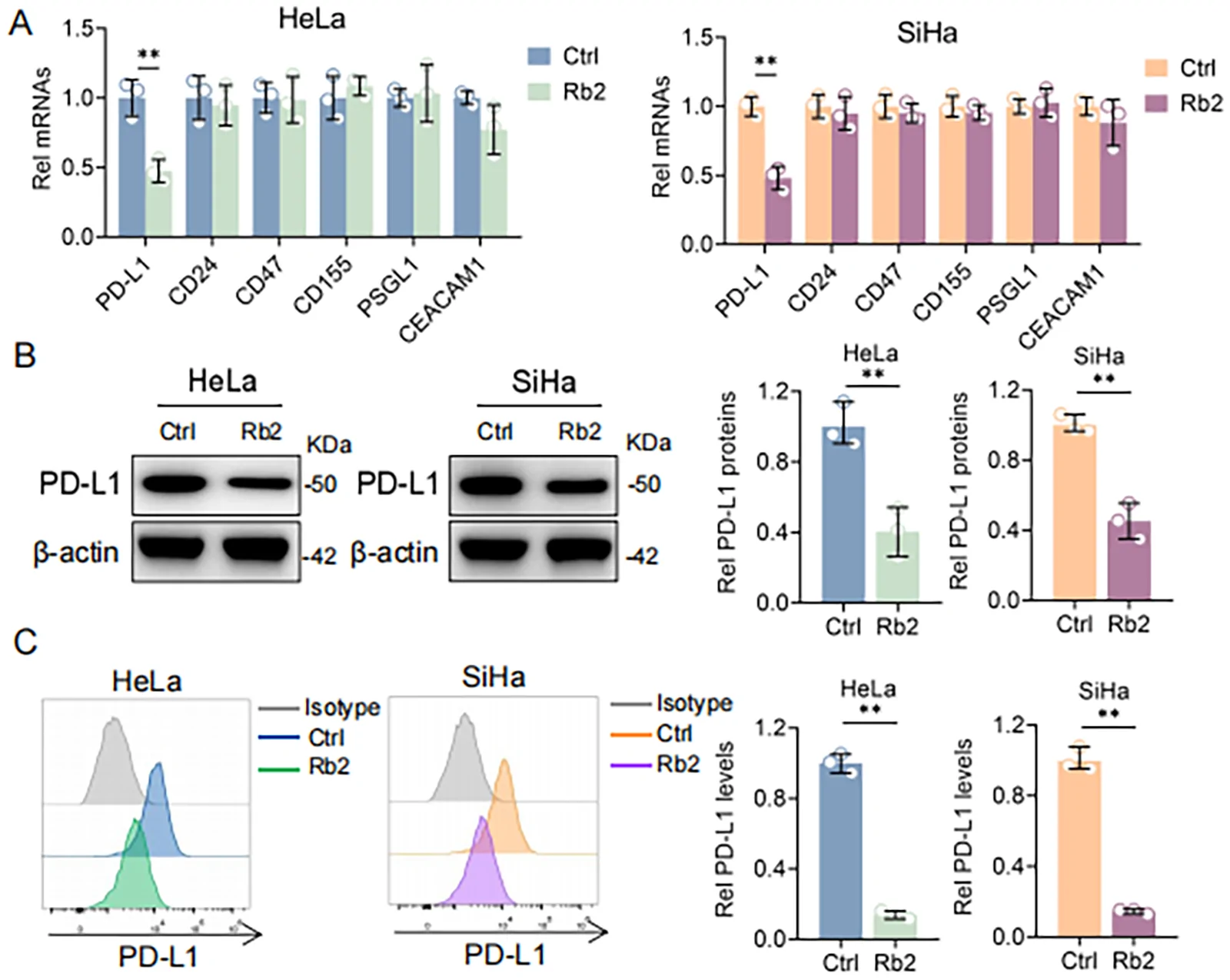
Ginsenoside Rb2 downregulates PD-L1 expression in cervical cancer cell. **(A)** RT-qPCR analysis of immune checkpoint-related genes, including PD-L1, CD24, CD47, CD155, PSGL1, and CEACAM1, in HeLa and SiHa cells treated with Rb2. **(B)** Western blot analysis of PD-L1 protein expression in HeLa and SiHa cells following Rb2 treatment, with corresponding densitometric quantification. **(C)** Flow cytometric analysis of cell-surface PD-L1 expression in HeLa and SiHa cells treated with Rb2. Representative histograms and quantification are shown. Data are presented as mean ± SD from three independent biological replicates (n = 3). **P < 0.01.

### Ginsenoside Rb2 suppresses glycolysis-mediated histone lactylation and PD-L1 transcription in cervical cancer cells

Previous studies have shown that cervical cancer growth is highly dependent on the Warburg effect, which is characterized by enhanced glycolysis and lactate production (30). To determine whether Rb2 affects cervical cancer progression through metabolic reprogramming, Seahorse extracellular flux analysis was performed. In both HeLa and SiHa cells, Rb2 treatment significantly reduced glycolytic activity and glycolytic capacity, as indicated by decreased ECAR values (**Figure 3A**). As the end product of glycolysis, lactate was once regarded merely as a metabolic waste product. However, emerging evidence has revealed that lactate can serve as a substrate for histone lactylation, thereby influencing gene transcription (31). Accordingly, we measured intracellular lactate levels and global histone lactylation. Rb2 treatment markedly reduced lactate production in cervical cancer cells and led to a significant decrease in pan-lactylation (Pan-Kla) levels (**Figures 3B, C**). To further characterize the specific histone lactylation sites affected by Rb2, we examined several established lactylation marks, including H3K9la, H3K18la, H3K23la, H4K5la, H4K8la, H4K12la, and H4K16la. Among these, Rb2 selectively decreased H3K18la and H4K12la, while the other sites remained largely unchanged (**Figures 3D, E**). Because H3K18la and H4K12la are well-recognized histone lactylation marks associated with active gene transcription (32), we further examined whether these modifications regulate PD-L1 transcription using ChIP-qPCR. Rb2 treatment significantly reduced the enrichment of H3K18la and H4K12la at the PD-L1 promoter in cervical cancer cells; notably, the inhibitory effect on H3K18la-associated transcriptional regulation appeared more pronounced than that on H4K12la. In addition, H3K18la can significantly regulate the transcription of the classical oncogene c-Myc (33), while H4K12la does not have this function (**Figures 3F, G**). These results indicate that H3K18la, rather than H4K12la, is the dominant lactylation mark mediating Rb2-dependent transcriptional regulation at the PD-L1 and c-Myc promoters, whereas H4K12la contributes to PD-L1 regulation to a lesser extent and is not involved in c-Myc transcription. Together, these data suggest that Rb2 suppresses PD-L1 transcription by inhibiting glycolysis-driven lactate production and reducing H3K18la- and H4K12la-mediated histone lactylation, thereby restraining the malignant progression of cervical cancer.

**Figure 3:**
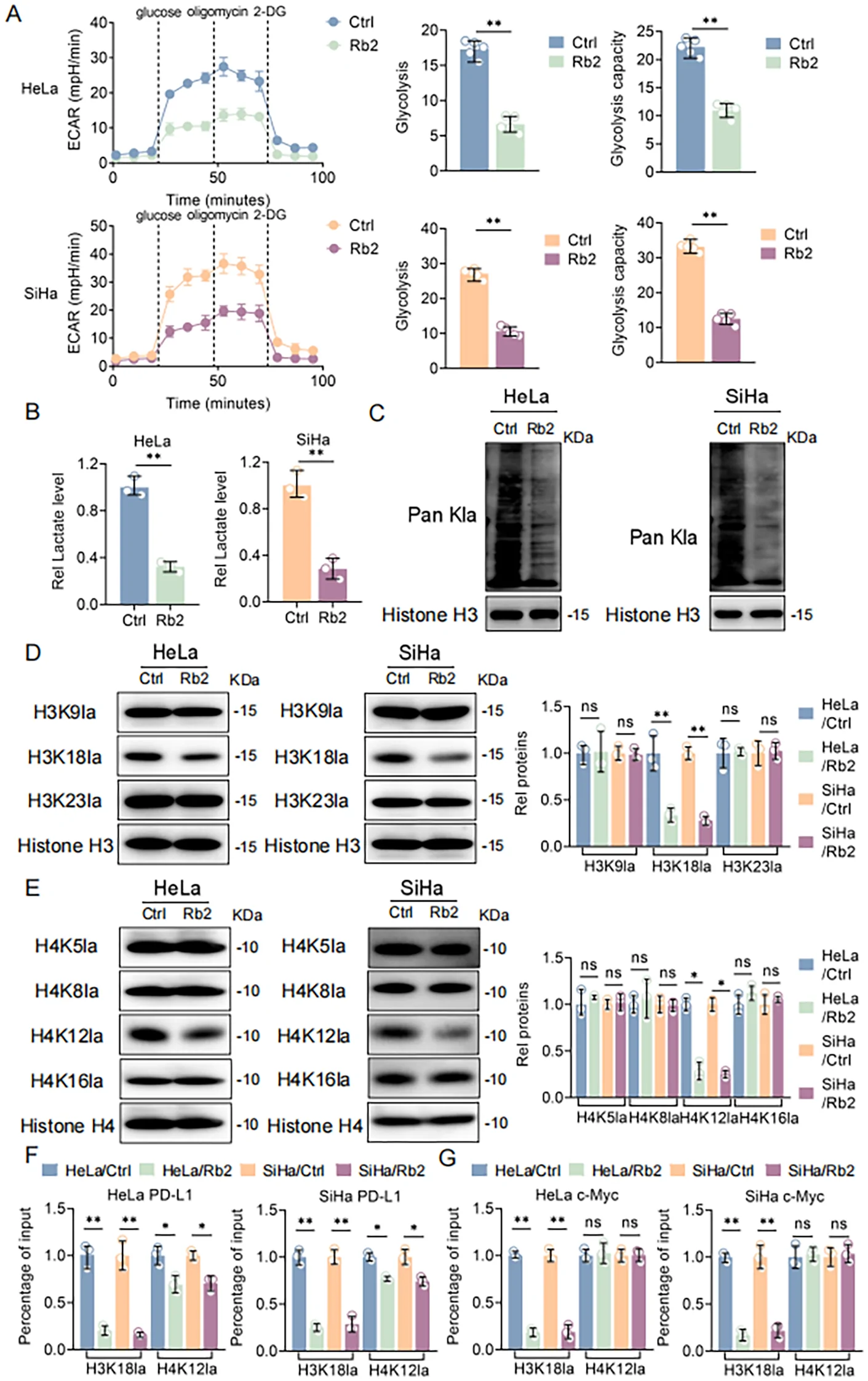
Ginsenoside Rb2 suppresses glycolysis-mediated histone lactylation and PD-L1 transcription in cervical cancer cells. **(A)** Extracellular acidification rate (ECAR) analysis of HeLa and SiHa cells treated with Rb2. Glycolysis and glycolytic capacity were quantified as indicated. **(B)** Relative intracellular lactate levels in HeLa and SiHa cells following Rb2 treatment. **(C)** Western blot analysis of total histone lactylation in histone extracts from HeLa and SiHa cells after Rb2 treatment. **(D)** Western blot analysis of site-specific histone lactylation marks, including H3K9la, H3K18la, H3K23la in HeLa and SiHa cells with or without Rb2 treatment. **(E)** Western blot analysis of site-specific histone lactylation marks, including H4K5la, H4K8la, H4K12la, and H4K16la, in HeLa and SiHa cells with or without Rb2 treatment. **(F)** ChIP-qPCR analysis showing the enrichment of H3K18la and H4K12la at the PD-L1 promoter regions in HeLa and SiHa cells following Rb2 treatment. **(G)** ChIP-qPCR analysis showing the enrichment of H3K18la and H4K12la at the c-Myc promoter regions in HeLa and SiHa cells following Rb2 treatment. Data are presented as mean ± SD from three independent biological replicates (n = 3). *P < 0.05, **P < 0.01; ns, not significant.

### Ginsenoside Rb2 selectively inhibits LDHA protein expression

To clarify how Rb2 alters the glycolytic program in cervical cancer cells, we examined the expression of key glycolytic enzymes. Semi-quantitative Western blot analysis revealed that among the major glycolytic enzymes tested, including HK1, HK2, PFKM, PFKL, PFKP, PKM1, PKM2, LDHA, and LDHB, only LDHA protein expression was markedly decreased following Rb2 treatment, whereas the others showed no obvious changes (**Figure 4A**). To determine whether Rb2 regulates these metabolic enzymes at the transcriptional level, RT-qPCR analysis was performed. The results showed that Rb2 did not significantly alter the mRNA levels of these glycolytic enzymes, including LDHA (**Figure 4B**). In addition, treatment of cervical cancer cells with increasing concentrations of Rb2 further confirmed that LDHA protein expression decreased in a dose-dependent manner (**Figure 4C**). These findings indicate that Rb2 selectively downregulates LDHA at the protein level rather than the transcriptional level, suggesting that LDHA may serve as a critical metabolic target through which Rb2 modulates glycolytic reprogramming, histone lactylation, and cervical cancer progression.

**Figure 4:**
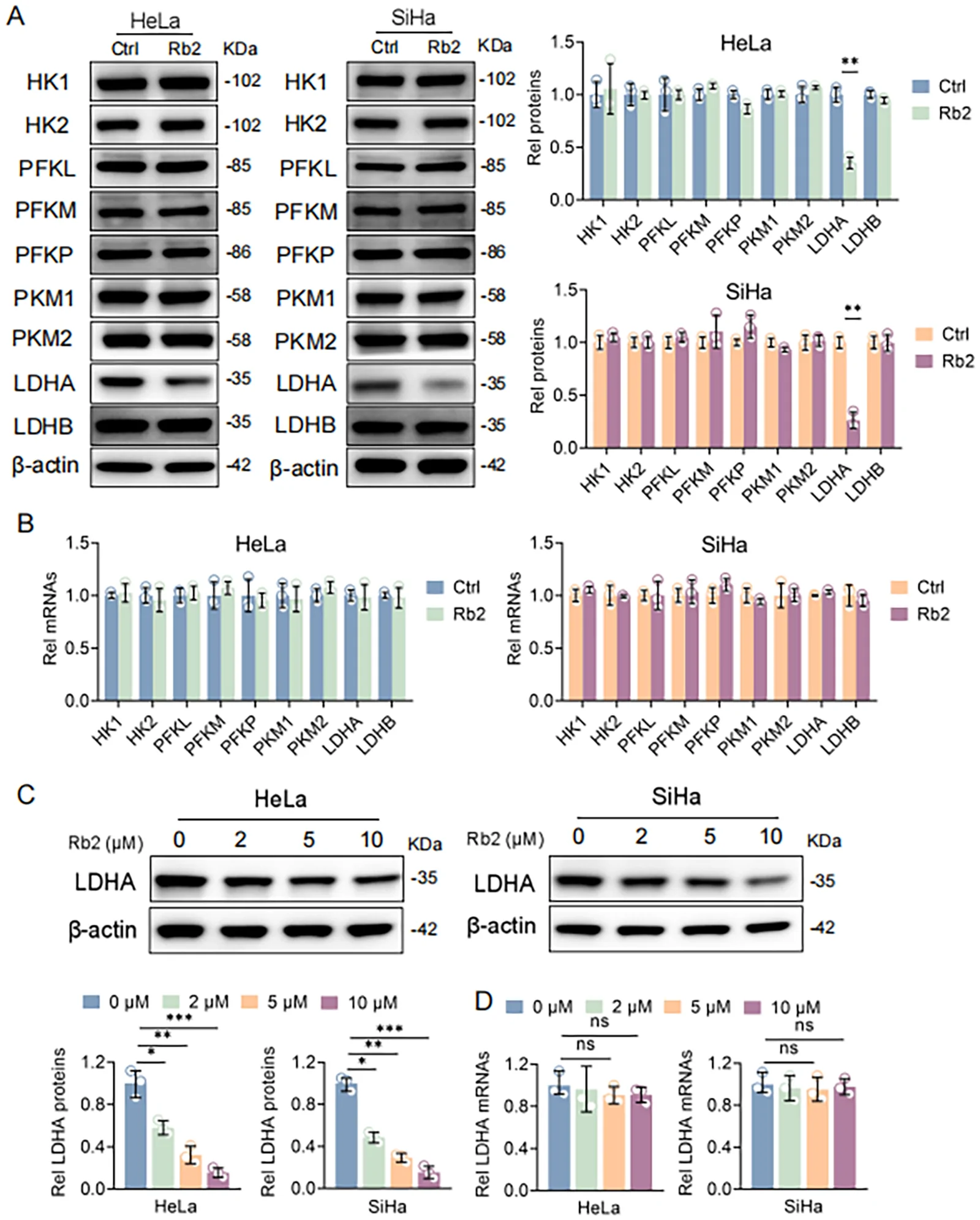
Ginsenoside Rb2 selectively inhibits LDHA protein expression. **(A)** Western blot screening of major glycolysis-related enzymes in HeLa and SiHa cells after Rb2 treatment, including HK1, HK2, PFKL, PFKM, PFKP, PKM1, PKM2, LDHA, and LDHB, with corresponding quantification. **(B)** RT-qPCR analysis of the corresponding glycolysis-related genes in HeLa and SiHa cells treated with Rb2. Relative mRNA levels are shown. **(C)** Western blot analysis of LDHA protein expression in HeLa and SiHa cells treated with increasing concentrations of Rb2 (0, 2, 5, and 10 μM). Quantification of relative LDHA protein levels is shown below. **(D)** RT-qPCR analysis of LDHA mRNA expression in HeLa and SiHa cells treated with increasing concentrations of Rb2 (0, 2, 5, and 10 μM). Data are presented as mean ± SD from three independent biological replicates (n = 3). *P < 0.05, **P < 0.01; ns, not significant.

### Rb2 inhibits PD-L1 expression through LDHA

Lactate dehydrogenase A (LDHA), a key rate-limiting enzyme in glycolysis, catalyzes the conversion of pyruvate to lactate in the final step of the glycolytic pathway and plays a central role in the Warburg effect (18). To further determine whether LDHA mediates the effects of Rb2 on glycolysis and lactate production, LDHA was overexpressed in cervical cancer cells, and the overexpression efficiency was confirmed by Western blotting (**Figure 5A**). Subsequent rescue experiments showed that enforced LDHA expression markedly attenuated the inhibitory effects of Rb2. Specifically, the Rb2-induced reductions in PD-L1 expression, H3K18la level, lactate production, and cell viability were all partially reversed by LDHA overexpression (**Figures 5B–F**). Taken together, these data demonstrate that LDHA is a key functional mediator of Rb2 activity, and that restoration of LDHA expression substantially rescues the antitumor effects induced by Rb2.

**Figure 5:**
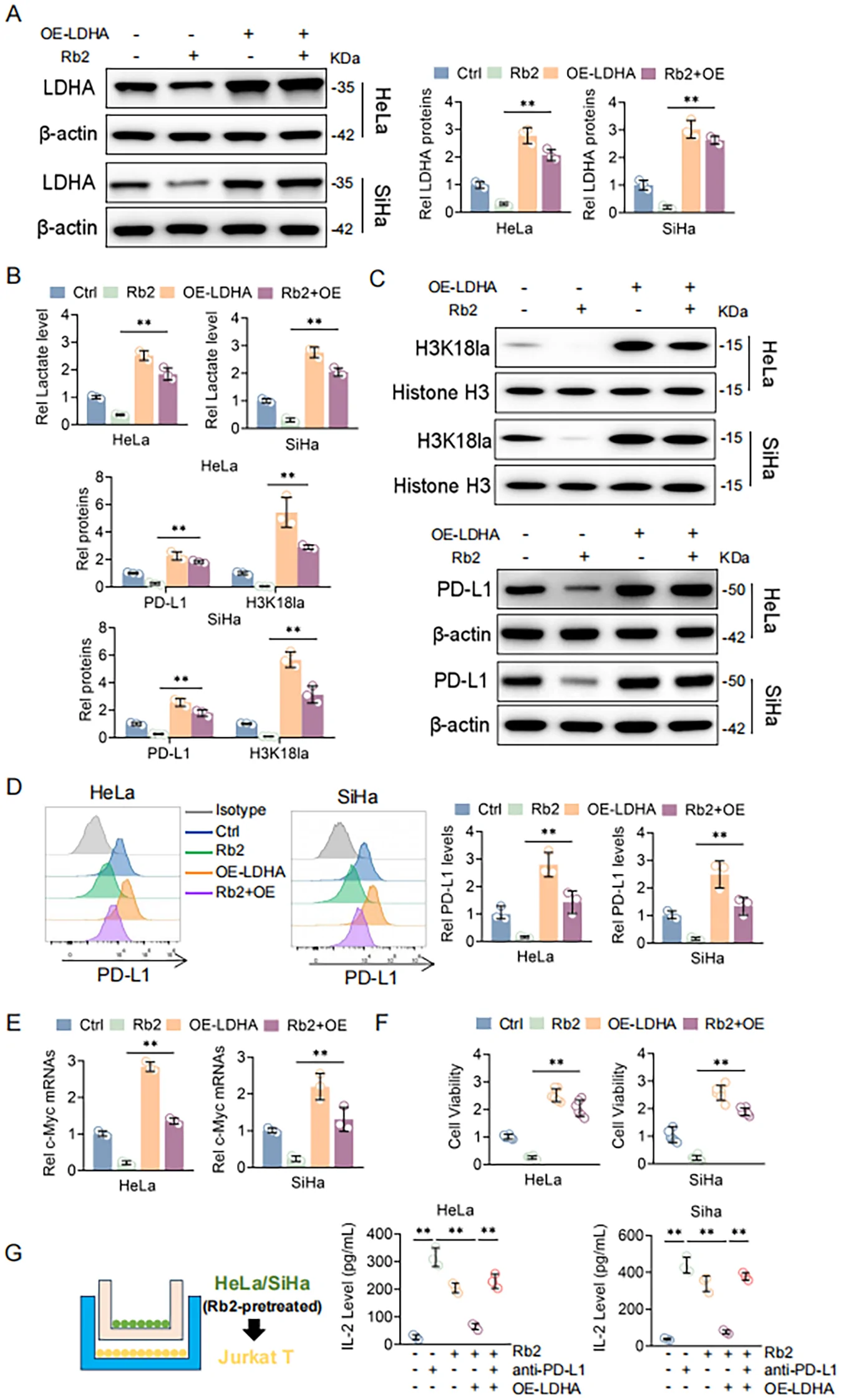
Rb2 inhibits PD-L1 expression through LDHA. **(A)** Western blot analysis of LDHA protein expression in HeLa and SiHa cells treated with Rb2, LDHA overexpression (OE-LDHA), or the combination of Rb2 and OE-LDHA. Quantification of relative LDHA protein levels is shown on the right. **(B)** Intracellular lactate levels in HeLa and SiHa cells under the indicated treatments. **(C)** Western blot analysis of H3K18la and PD-L1 expression in HeLa and SiHa cells treated with/without Rb2 and OE-LDHA. Quantification of relative PD-L1 and H3K18la protein levels is shown below. **(D)** Flow cytometric analysis of cell-surface PD-L1 expression in HeLa and SiHa cells under the indicated conditions. **(E)** RT-qPCR analysis of c-Myc mRNA expression in HeLa and SiHa cells under the indicated treatments. **(F)** Cell viability of HeLa and SiHa cells under the indicated treatments. **(G)** Jurkat T cells were co-cultured with control or Rb2-pretreated HeLa/SiHa cells (with or without LDHA overexpression) at a tumor-to-T-cell ratio of 1:10 for 24 h, in the presence or absence of a PD-L1 neutralizing antibody. IL-2 levels in the culture supernatant were quantified by ELISA. Data are presented as mean ± SD from three independent biological replicates (n = 3). **P < 0.01; ns, not significant.

To determine whether Rb2-mediated PD-L1 downregulation restores T-cell function, Jurkat T cells were co-cultured with Rb2-pretreated or control cervical cancer cells (**Figure 5G**). Co-culture with untreated tumor cells markedly suppressed IL-2 production by Jurkat cells, whereas both PD-L1 neutralizing antibody and Rb2 pretreatment significantly restored IL-2 levels to a comparable extent. Notably, LDHA overexpression abolished the Rb2-mediated IL-2 restoration, and this effect was again rescued by PD-L1 blockade, indicating that LDHA regulates T-cell function through PD-L1-dependent mechanisms. These results provide direct functional evidence that Rb2 enhances T-cell activation through the LDHA/PD-L1 axis.

### Ginsenoside Rb2 promotes LDHA degradation through the autophagy–lysosome pathway by reducing LDHA protein stability

To further explore the mechanism by which Rb2 suppresses LDHA protein expression, we assessed the stability of LDHA at both the protein and mRNA levels. Cervical cancer cells were treated with cycloheximide (CHX) or actinomycin D (ActD) to block protein or mRNA synthesis (34), respectively, and LDHA degradation was monitored over time (0, 3, 6, and 12 h) in the presence of Rb2. The results showed that Rb2 significantly accelerated LDHA protein degradation, whereas it did not affect the stability of LDHA mRNA (**Figures 6A, B**). Because protein stability is primarily regulated by either the ubiquitin–proteasome system or the autophagy-lysosome pathway, we next treated Rb2-exposed cervical cancer cells with the proteasome inhibitor MG132 or the lysosomal inhibitor chloroquine (CQ) (34). Inhibition of lysosomal function, but not proteasomal activity, markedly rescued LDHA protein levels, suggesting that Rb2 promotes LDHA degradation mainly through the autophagy–lysosome pathway (**Figures 6C, D**). To further confirm the lysosomal localization of LDHA, lysosome immunoprecipitation (LysoIP) assays were performed. LDHA was detectable in isolated lysosomal fractions, and its lysosomal accumulation was markedly increased after EBSS treatment, indicating that LDHA can indeed enter lysosomes for degradation (**Figure 6E**). Together, these findings show that Rb2 decreases LDHA protein stability by promoting its autophagy–lysosome-dependent degradation, thereby contributing to the suppression of cervical cancer progression.

**Figure 6:**
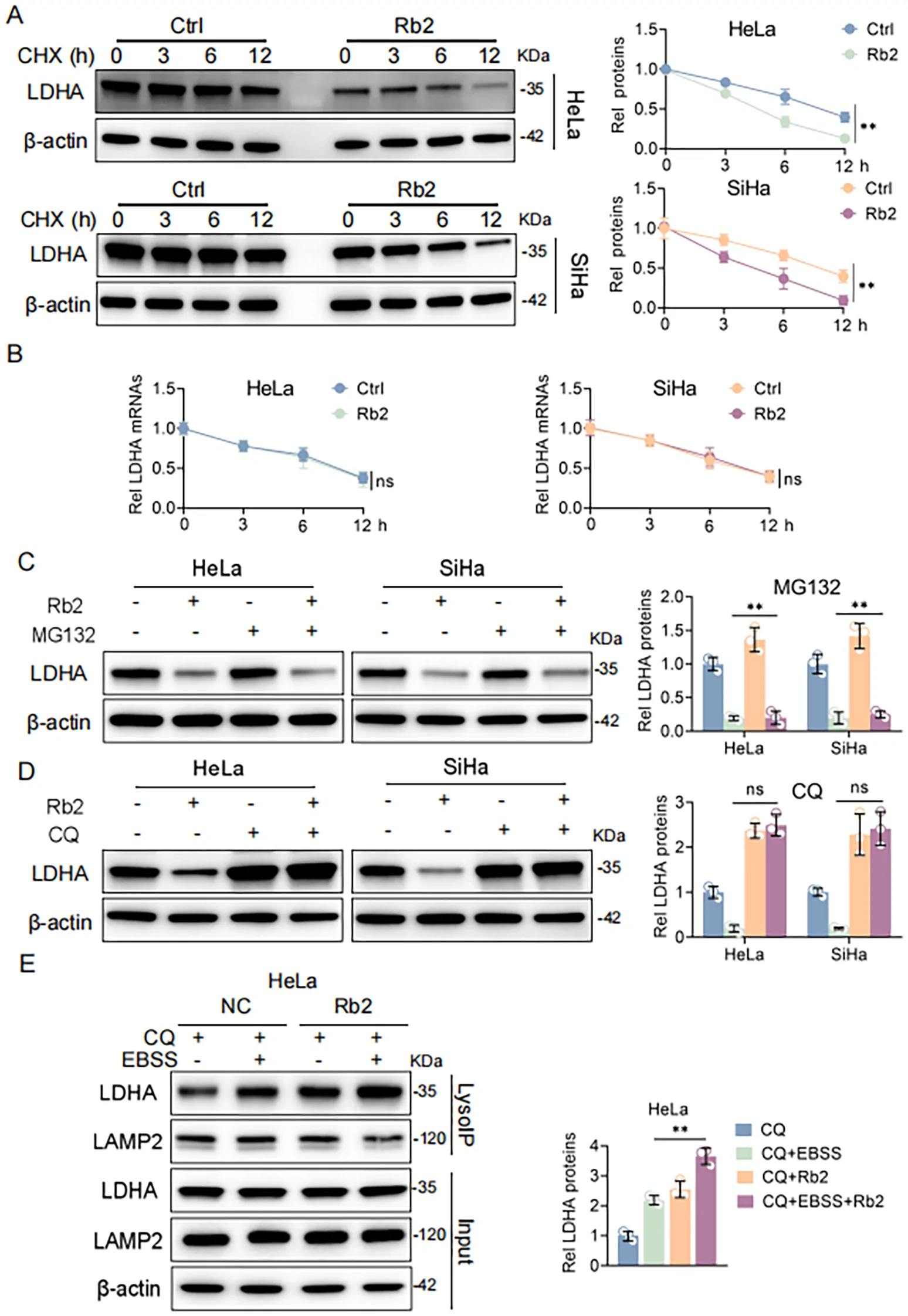
Ginsenoside Rb2 promotes LDHA degradation through the autophagy–lysosome pathway by reducing LDHA protein stability. **(A)** Cycloheximide (CHX) chase assay showing LDHA protein stability in HeLa and SiHa cells treated with or without Rb2. **(B)** RT-qPCR analysis of LDHA mRNA levels in HeLa and SiHa cells treated with Rb2 for the indicated times. **(C)** Western blot analysis of LDHA protein expression in HeLa and SiHa cells treated with Rb2 in the presence or absence of the proteasome inhibitor MG132. **(D)** Western blot analysis of LDHA protein expression in HeLa and SiHa cells treated with Rb2 in the presence or absence of the lysosomal inhibitor chloroquine (CQ). **(E)** Lysosome immunoprecipitation (LysoIP) assay in HeLa cells showing the accumulation of LDHA in lysosomes under the indicated treatments. Data are presented as mean ± SD from three independent biological replicates (n = 3). **P < 0.01; ns, not significant.

### Ginsenoside Rb2 promotes p62-mediated autophagic–lysosomal degradation of LDHA

p62/SQSTM1 is a key adaptor protein in selective autophagy that facilitates the delivery of ubiquitinated cargo to autophagosomes and promotes subsequent lysosomal degradation (35). We first examined whether Rb2 affects p62 expression and found that p62 protein levels were not significantly altered after Rb2 treatment in cervical cancer cells (**Figure 7A**). To further determine whether p62 is involved in Rb2-induced LDHA degradation, gain- and loss-of-function experiments were performed. Overexpression of p62 in the presence of Rb2 led to a more pronounced reduction in LDHA protein levels, whereas p62 knockdown significantly reversed the Rb2-induced decrease in LDHA expression (**Figures 7B, C**). We next examined whether Rb2 regulates the interaction between p62 and LDHA. Co-immunoprecipitation assays revealed that Rb2 treatment enhanced the binding between endogenous p62 and LDHA in cervical cancer cells. Consistently, in HEK-293T cells ectopically expressing Flag-LDHA and His-p62, Rb2 also strengthened the interaction between exogenous p62 and LDHA (**Figures 7D, E**). Taken together, these results indicate that Rb2 facilitates p62-mediated autophagic–lysosomal degradation of LDHA by enhancing the association between p62 and LDHA.

**Figure 7:**
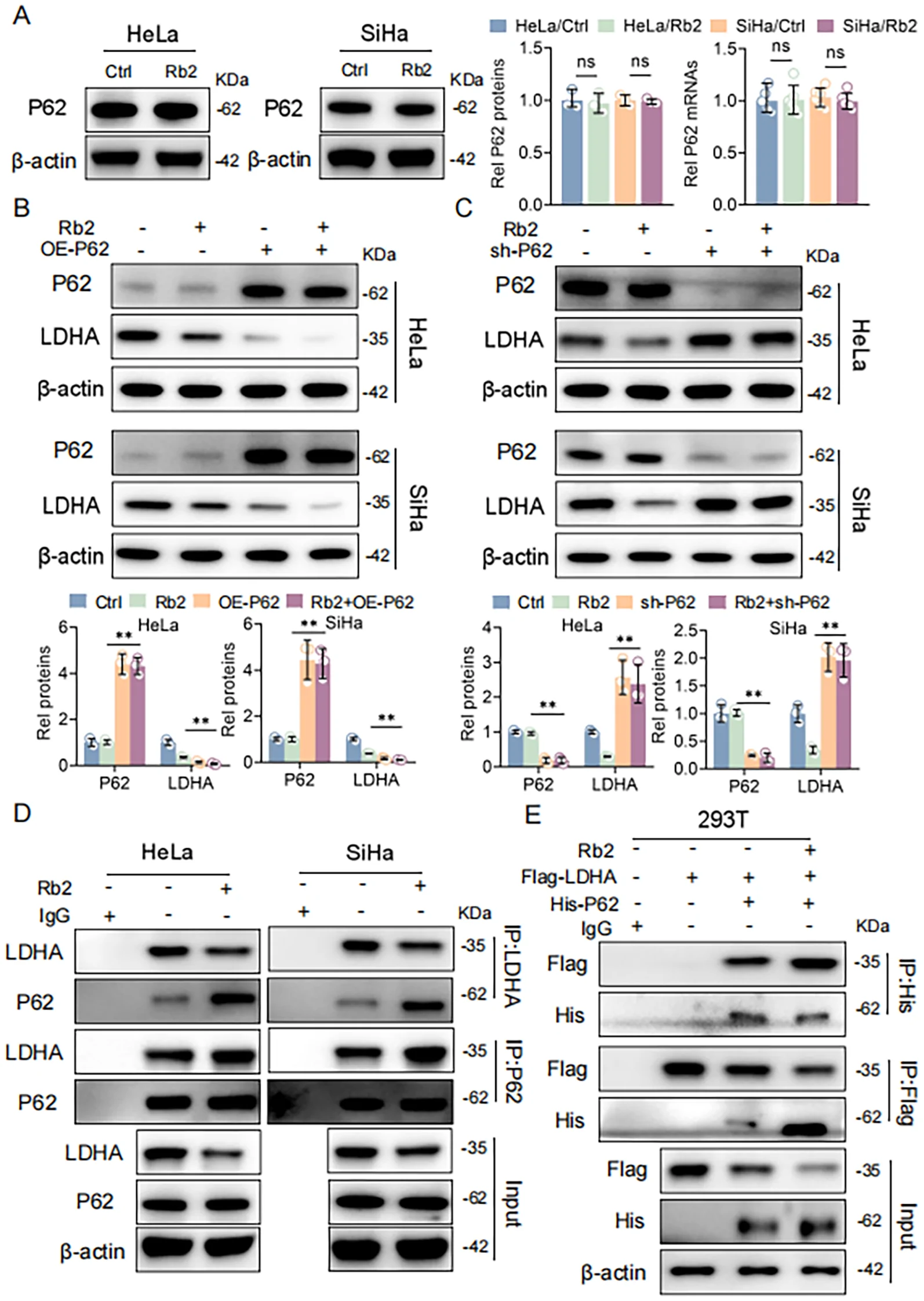
Ginsenoside Rb2 promotes p62-mediated autophagic–lysosomal degradation of LDHA. **(A)** Western blot and RT-qPCR analyses of p62 expression in HeLa and SiHa cells treated with or without Rb2. **(B)** Western blot analysis of p62 and LDHA protein expression in HeLa and SiHa cells transfected with p62 overexpression plasmid (OE-p62) in the presence or absence of Rb2. **(C)** Western blot analysis of p62 and LDHA protein expression in HeLa and SiHa cells transfected with p62 knockdown plasmid (sh-p62) in the presence or absence of Rb2. Quantification of relative protein levels is shown below. **(D)** Co-immunoprecipitation (Co-IP) assays showing the interaction between endogenous p62 and LDHA in HeLa and SiHa cells treated with or without Rb2. Immunoprecipitation was performed using antibodies against LDHA or p62, followed by immunoblotting for the indicated proteins. IgG was used as a negative control. **(E)** Co-IP assays in 293T cells co-transfected with Flag-LDHA and His-p62, with or without Rb2 treatment. Immunoprecipitation was performed using anti-His or anti-Flag antibodies, followed by immunoblotting with the indicated antibodies to detect the interaction between exogenous p62 and LDHA. Data are presented as mean ± SD from three independent biological replicates (n = 3). **P < 0.01; ns, not significant.

### Ginsenoside Rb2 directly associates with both p62 and LDHA and enhances the p62-LDHA interaction

To further elucidate the mechanism by which Rb2 regulates p62 and LDHA, we explored the potential physical interactions among Rb2, p62, and LDHA by molecular docking analysis. Structural simulations showed that Rb2 could form multiple hydrogen bonds with both LDHA and p62, with favorable binding affinities. Specifically, Rb2 was predicted to interact with LDHA at ASN-163, ARG-170, LYS-244, SER-248, TRP-249, ALA-250, and VAL-269 (binding energy, -9.0 kcal/mol), and with p62 at ASN-120, SER-152, LYS-165, and ARG-161 (binding energy, -6.7 kcal/mol) (**Figures 8A, B**). To validate these predicted interactions experimentally, biotinylated Rb2 (Bio-Rb2) was synthesized (**Figure 8C**). Competitive pull-down assays confirmed the specificity of Bio-Rb2: pre-incubation of cell lysates with unlabeled free Rb2 at increasing molar ratios (1:5 and 1:10, Bio-Rb2:free Rb2) dose-dependently competed with Bio-Rb2 for binding to both LDHA and p62, indicating that biotinylation did not impair the target-recognition capacity of Rb2 (**Figure 8C**). We then performed Bio-Rb2 pull-down assays with cervical cancer cell lysates. Bio-Rb2, but not free Rb2 or beads alone, pulled down both p62 and LDHA in both HeLa and SiHa cells, indicating that Rb2 can associate with both proteins (**Figures 8D, E**). Notably, p62 overexpression further enhanced the interaction between Rb2 and p62 (**Figures 8F, G**). These findings support the notion that Rb2 can bind both p62 and LDHA, thereby facilitating the p62-dependent autophagic–lysosomal degradation of LDHA.

**Figure 8:**
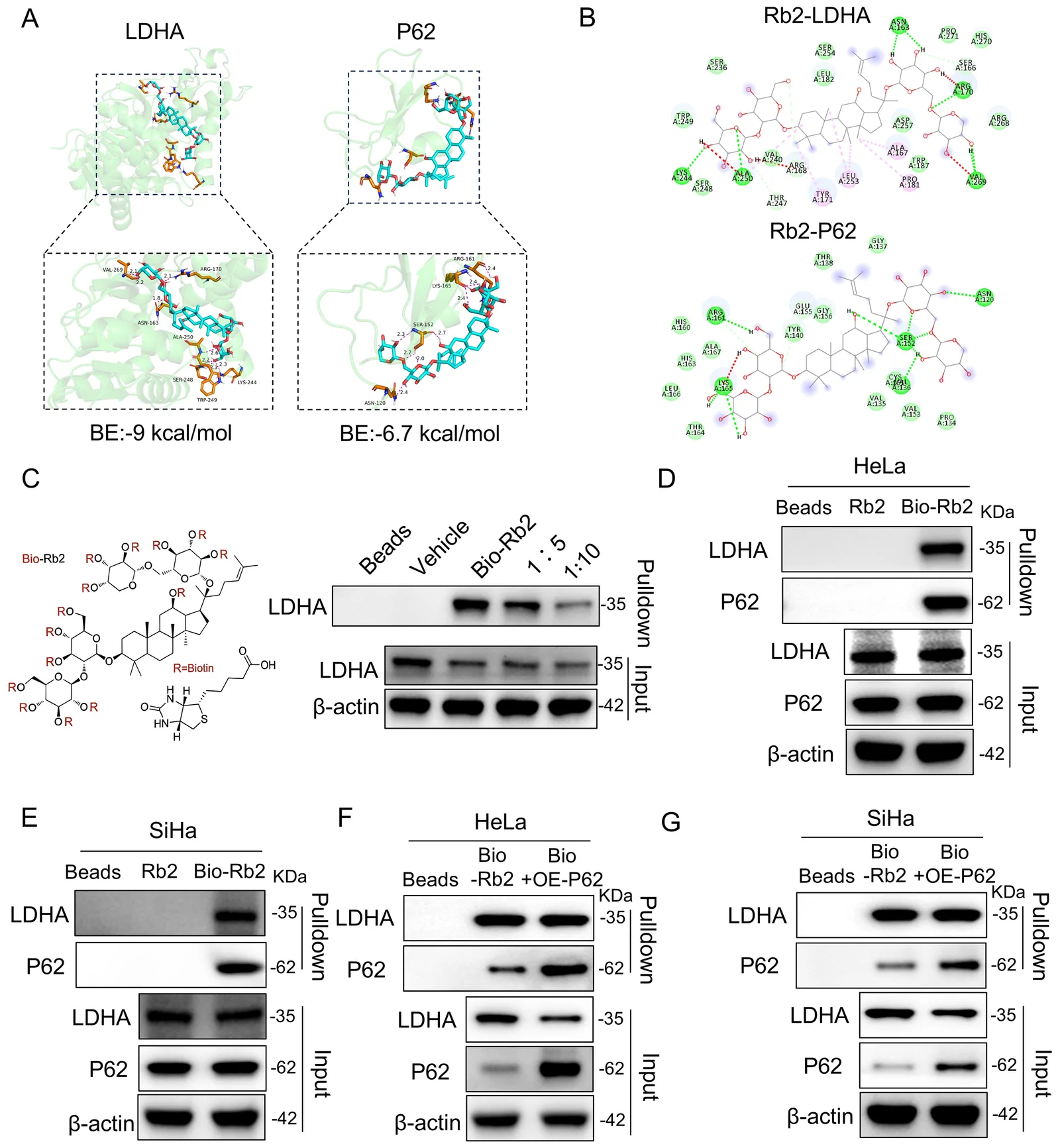
Ginsenoside Rb2 directly associates with both p62 and LDHA and enhances the p62–LDHA interaction. **(A)** Molecular docking analysis showing the predicted binding modes of Rb2 with LDHA and p62. **(B)** Two-dimensional interaction diagrams illustrating the predicted molecular interactions between Rb2 and LDHA or p62. **(C)** Chemical structure of biotinylated Rb2 (Bio-Rb2) (left) and competitive pull-down assay showing that unlabeled free Rb2 dose-dependently competes with Bio-Rb2 for binding to LDHA and p62 at molar ratios of 1:5 and 1:10 (Bio-Rb2:free Rb2) (right). **(D)** Bio-Rb2 pull-down assay in HeLa cells showing the association of endogenous LDHA and p62 with Bio-Rb2. **(E)** Bio-Rb2 pull-down assay in SiHa cells showing the association of endogenous LDHA and p62 with Bio-Rb2. **(F)** Bio-Rb2 pull-down assay in HeLa cells transfected with OE-p62. **(G)** Bio-Rb2 pull-down assay in SiHa cells transfected with OE-p62. Data are presented as mean ± SD from three independent biological replicates (n = 3).

### Rb2 suppresses cervical cancer progression through LDHA *in vivo*

To determine whether LDHA overexpression could rescue the antitumor effects of Rb2 *in vivo*, we established a C57BL/6 mouse xenograft model using cervical cancer cells with or without LDHA overexpression. As expected, Rb2 treatment significantly reduced tumor size and tumor volume, whereas LDHA overexpression markedly attenuated the tumor-suppressive effect of Rb2 (**Figure 9A**). We further performed immunohistochemical staining on xenograft tumor tissues. The results showed that Rb2 significantly decreased the expression of H3K18la, PD-L1, and c-Myc, whereas LDHA overexpression substantially reversed these inhibitory effects (**Figure 9B**). Finally, to assess the clinical relevance of these findings, immunohistochemical analysis was performed on cervical cancer tissue specimens collected from patients. We found that patients with high LDHA expression also exhibited elevated PD-L1 expression, and correlation analysis demonstrated a significant positive association between LDHA and PD-L1 levels. Collectively, these *in vivo* and clinical data further support a model in which Rb2 suppresses cervical cancer progression by targeting the LDHA/histone lactylation/PD-L1 axis.

**Figure 9:**
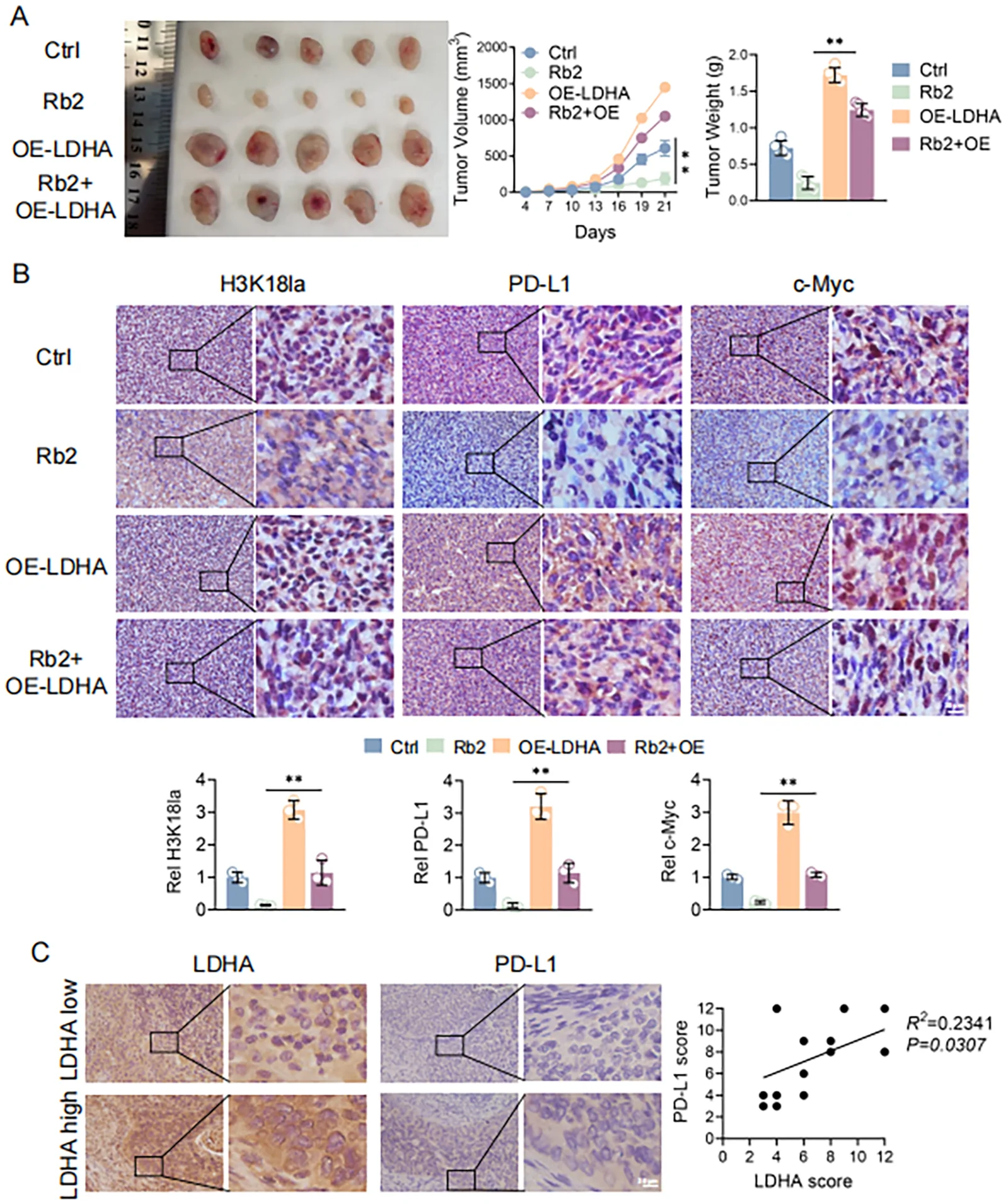
Rb2 suppresses cervical cancer progression through LDHA *in vivo*. **(A)** Representative images of xenograft tumors derived from U14 cells in the Ctrl, Rb2, OE-LDHA, and Rb2 + OE-LDHA groups. Tumor growth curves and final tumor weights are shown on the right. **(B)** Immunohistochemical staining of H3K18la, PD-L1, and c-Myc in xenograft tumor tissues from the indicated groups. **(C)** Representative immunohistochemical images of LDHA and PD-L1 expression in cervical cancer tissues with low or high LDHA expression. Correlation analysis showed that LDHA expression was positively associated with PD-L1 expression in cervical cancer specimens. Data are presented as mean ± SD (n = 6 mice per group). **P < 0.01.

## Discussion

In the present study, we identified a molecular cascade by which ginsenoside Rb2 suppresses cervical cancer progression. Mechanistically, Rb2 did not broadly inhibit all glycolytic enzymes, but selectively reduced LDHA protein abundance through a post-translational mechanism. Specifically, Rb2 enhanced the interaction between LDHA and the selective autophagy adaptor p62/SQSTM1, thereby promoting autophagy–lysosome-dependent degradation of LDHA. Loss of LDHA led to reduced lactate production and attenuation of glycolysis-driven histone lactylation, particularly at H3K18la and H4K12la. Decreased enrichment of these lactylation marks at the PD-L1 promoter was accompanied by transcriptional repression of PD-L1, ultimately resulting in inhibition of cervical cancer cell proliferation, migration, invasion, and xenograft growth. Notably, rescue experiments demonstrated that restoration of LDHA substantially reversed the effects of Rb2 on lactate production, histone lactylation, PD-L1 expression, and tumor growth, establishing LDHA as a critical functional node in this pathway. In parallel, the positive correlation between LDHA and PD-L1 in cervical cancer tissues further supports the clinical relevance of the Rb2–p62–LDHA–lactylation-PD-L1 signaling axis. Collectively, these findings delineate an integrated metabolic-epigenetic-immune regulatory network through which Rb2 exerts antitumor effects in cervical cancer.

PD-L1 has emerged as a central mediator of tumor immune evasion and a major therapeutic target across multiple malignancies, including cervical cancer (36). Persistent HPV infection creates a chronically inflamed microenvironment in which cervical cancer cells frequently exploit the PD-1/PD-L1 axis to dampen antitumor T-cell responses, thereby facilitating immune escape and disease progression (37). This biological context provides a strong rationale for targeting PD-L1 in cervical cancer. Clinically, blockade of the PD-1/PD-L1 pathway has already demonstrated therapeutic benefit in several tumor types, such as melanoma, non-small cell lung cancer, renal cell carcinoma, urothelial carcinoma, hepatocellular carcinoma, triple-negative breast cancer, and recurrent or metastatic cervical cancer (38, 39). In cervical cancer in particular, anti-PD-1-based strategies have shown meaningful efficacy in selected patients, validating PD-L1 as a therapeutically actionable vulnerability (36, 40). However, the current treatment paradigm relies predominantly on monoclonal antibodies, and only a subset of patients achieve durable responses, while immune-related adverse events, high cost, and acquired resistance remain major challenges (10). These limitations underscore the need to identify alternative strategies that can reduce PD-L1 expression or function upstream of checkpoint engagement. By demonstrating that Rb2 decreases PD-L1 transcription through metabolic and epigenetic remodeling, this study expands the conceptual framework for PD-L1-directed intervention in cervical cancer.

A major conceptual advance of this work is the identification of an LDHA/histone lactylation/PD-L1 regulatory pathway in cervical cancer. PD-L1 expression is known to be regulated at multiple levels, including genomic alterations, inflammatory signaling, oncogenic pathways, epigenetic modifications, and post-translational mechanisms (41). Previous studies have shown that PD-L1 can be induced by DNA methylation, histone acetylation, histone methylation, microRNAs, and protein stabilization mechanisms have all been implicated in shaping PD-L1 abundance in tumor cells (42). Compared with these established regulatory routes, the involvement of histone lactylation in PD-L1 transcription remains much less explored, particularly in cervical cancer. Histone lactylation has only recently been recognized as a metabolically sensitive epigenetic mark that links lactate accumulation to gene activation (31). Our data suggest that Rb2 suppresses PD-L1 not through canonical inflammatory checkpoint signaling alone, but by selectively attenuating LDHA-dependent lactate generation and reducing H3K18la and H4K12la occupancy at the PD-L1 promoter. This mechanism is conceptually distinct from previously described approaches that target PD-L1 through cytokine signaling inhibition or direct checkpoint blockade, because it reveals that tumor metabolic flux can transcriptionally program immune checkpoint expression through site-specific histone lactylation. The observation that H3K18la appears more prominently involved than H4K12la further suggests that not all lactylation marks contribute equally to PD-L1 regulation. Mechanistically, our ChIP-qPCR data indicate that H4K12la is not functionally equivalent to H3K18la, because H4K12la was not involved in c-Myc transcription; the precise contribution of H4K12la to PD-L1 transcription, and its potential relationship with specific transcription factors, therefore remains to be defined and warrants mark-specific investigation. Thus, our study identifies a functional metabolic-epigenetic circuit that controls PD-L1 expression and highlights histone lactylation as a previously underappreciated layer of immune checkpoint regulation in cervical cancer.

Another important aspect of this study is that it reveals a noncanonical antitumor mode of action for a natural product by linking a traditional Chinese medicine-derived compound to selective autophagic control of immune checkpoint signaling (43). Current PD-L1-targeting strategies are still dominated by therapeutic antibodies or engineered biologics, which have transformed cancer treatment but are constrained by limited patient response rates, systemic immune toxicities, poor penetration into certain tumor niches, and substantial economic burden (44). Small-molecule or natural-compound-based modulators of PD-L1 are being actively explored because they may offer advantages in oral bioavailability, intracellular target accessibility, combinatorial flexibility, and cost-effectiveness, yet most available candidates remain in preclinical development and often lack mechanistic specificity (45). In parallel, Rb2 has been reported in previous studies to exert antitumor activities through inhibition of proliferation, induction of apoptosis (46). However, whether Rb2 can regulate immune checkpoints through coordinated metabolic and degradative mechanisms has remained largely unknown. Our findings extend the pharmacological repertoire of Rb2 by showing that it promotes p62-mediated autophagic–lysosomal degradation of LDHA, thereby suppressing lactate-dependent histone lactylation and ultimately reducing PD-L1 expression. From this perspective, Rb2 is not merely a general cytotoxic phytochemical; rather, it acts as a pathway-oriented regulator that integrates protein quality control, metabolic rewiring, epigenetic remodeling, and immune checkpoint suppression. Although the molecular basis underlying the Rb2-enhanced p62–LDHA interaction remains to be fully defined, our docking and pull-down data suggest that Rb2 may act as a molecular bridge that stabilizes the association between p62 and its cargo LDHA; whether post-translational modifications of p62 (e.g., phosphorylation) or conformational changes additionally contribute warrants further investigation.

Several limitations of this study should be acknowledged. First, although our co-culture experiments demonstrate that Rb2-mediated PD-L1 downregulation restores T-cell activation *in vitro*, the immunological consequences of PD-L1 reduction were not directly assessed *in vivo* in an intact immune system; functional analyses of tumor-infiltrating T cells in immunocompetent models will be required to fully establish the immune effects of Rb2. Second, while Bio-Rb2 pull-down, competitive pull-down, and molecular docking support an association between Rb2 and both p62 and LDHA, additional biophysical approaches such as surface plasmon resonance, microscale thermophoresis, CETSA, or DARTS, as well as chemical characterization of Bio-Rb2 by NMR/MS, would further strengthen the evidence for direct target engagement. Third, the precise molecular mechanism by which Rb2 enhances the p62–LDHA interaction (e.g., post-translational modifications of p62 or conformational changes) remains to be defined. Fourth, although H3K18la and H4K12la were identified as the principal lactylation marks affected in our system, the broader chromatin landscape and the possible involvement of additional lactylation-dependent transcriptional programs remain to be defined. Genome-wide epigenomic profiling (e.g., H3K18la ChIP-seq/CUT&Tag combined with RNA-seq) and dissection of the relative contribution of H4K12la will help clarify the full spectrum of genes regulated through this mechanism.

In summary, this study demonstrates a mechanism by which ginsenoside Rb2 suppresses cervical cancer progression. We show that Rb2 facilitates p62-dependent autophagic–lysosomal degradation of LDHA, leading to reduced lactate production, inhibition of H3K18la- and H4K12la-associated histone lactylation, transcriptional repression of PD-L1, and consequent suppression of malignant phenotypes. These findings not only establish a functional link between tumor metabolism, histone lactylation, and immune checkpoint regulation in cervical cancer, but also broaden the mechanistic understanding of how natural compounds can remodel oncogenic signaling networks. From a translational perspective, the identification of the LDHA/histone lactylation/PD-L1 axis provides a promising therapeutic framework and suggests that Rb2, or agents designed to mimic its mechanism, may serve as potential candidates for cervical cancer treatment, either alone or in combination with existing immunotherapeutic strategies.

## Data Availability

The original contributions presented in the study are included in the article/**Supplementary Material**. Further inquiries can be directed to the corresponding authors.
