# Supplementary material for: Ginsenoside Rb2 promotes p62-mediated LDHA autophagic degradation to inhibit H3K18la-dependent PD-L1 transcription in cervical cancer
Source: Front Oncol. 2026 Sep 28;16:1962784. doi: 10.3389/fonc.2026.1962784 (PMC13654782; doi:10.3389/fonc.2026.1962784)

Figure 2B

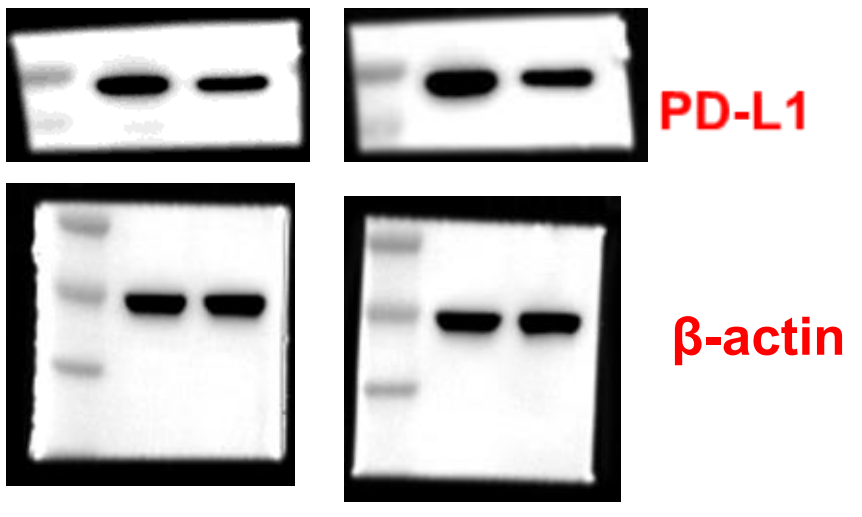

Figure 3C

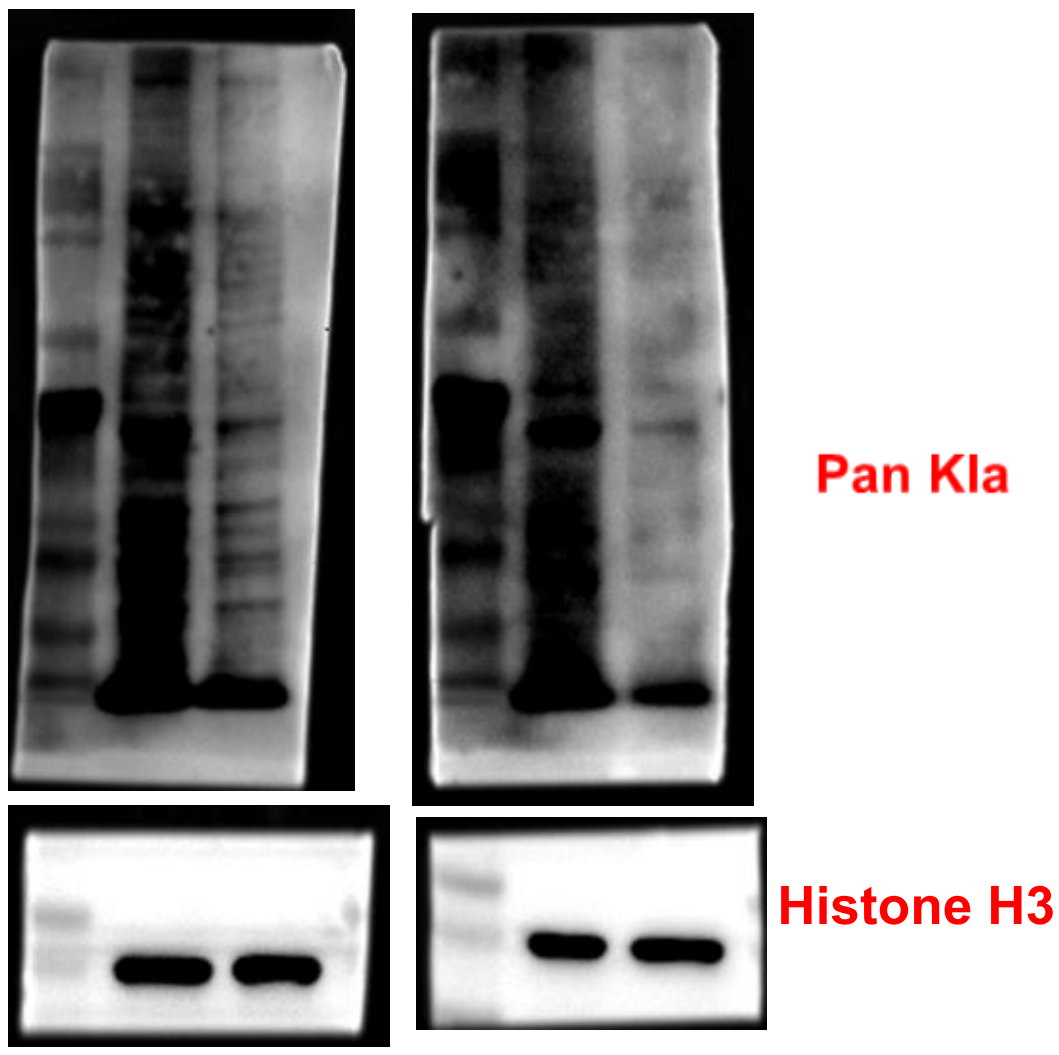

Figure 3D

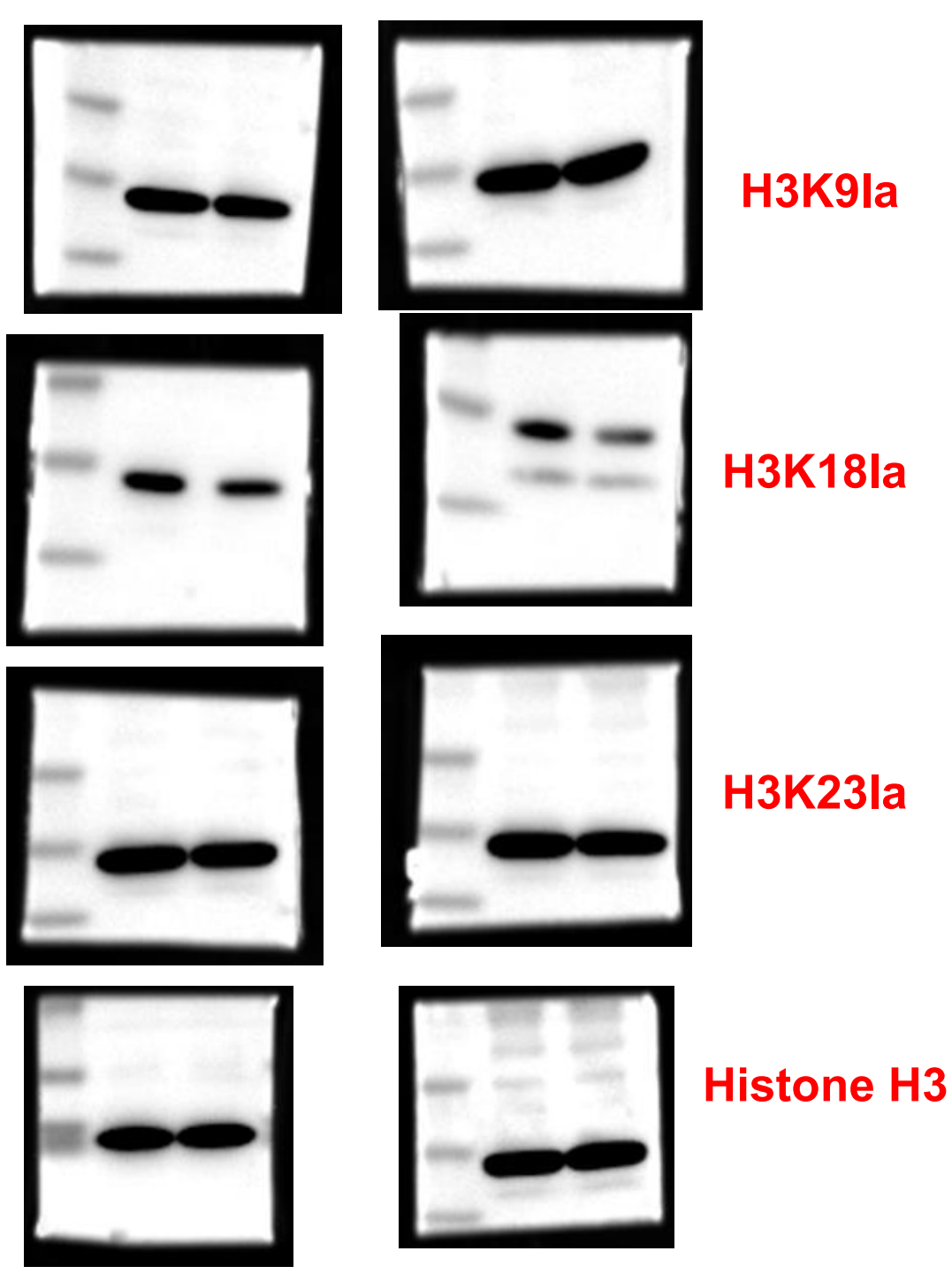

Figure 3E

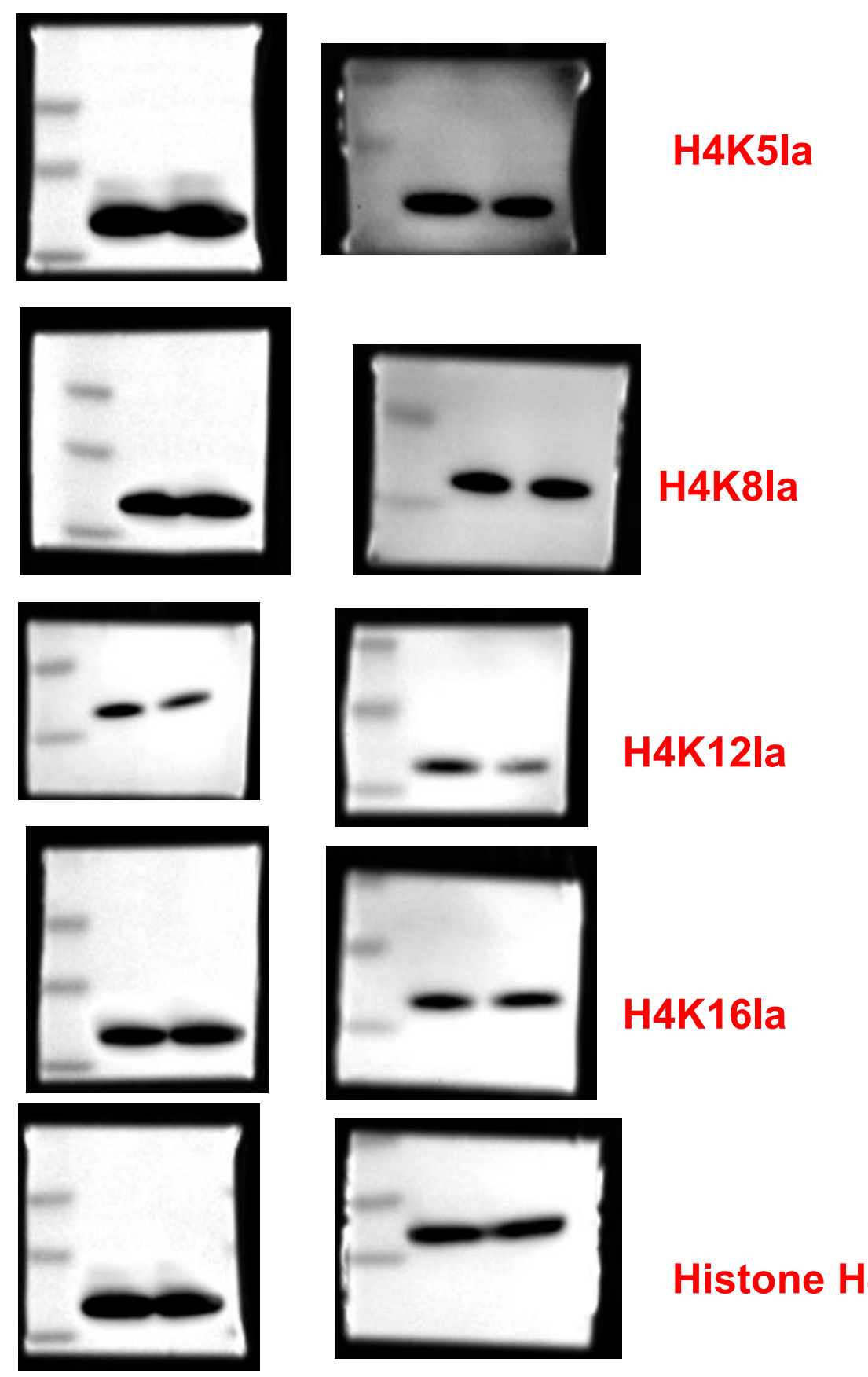

Figure 4A

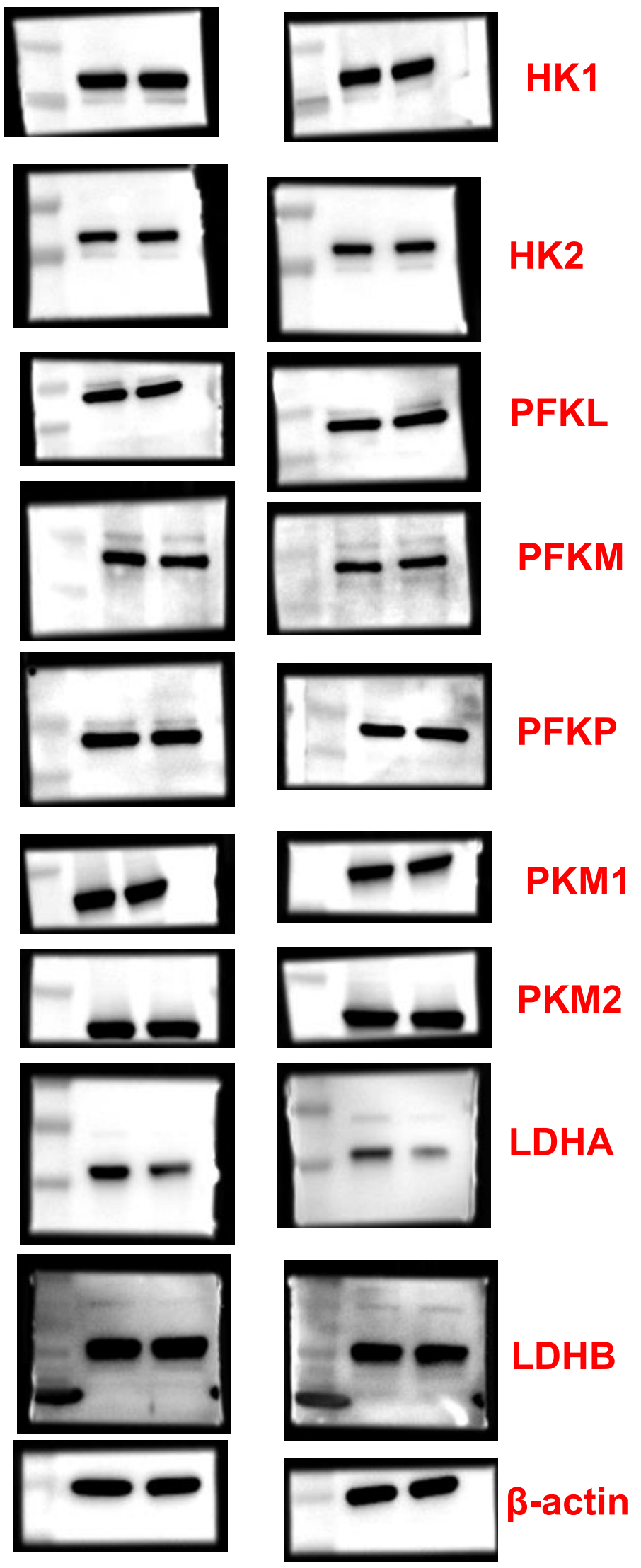

Figure 4C

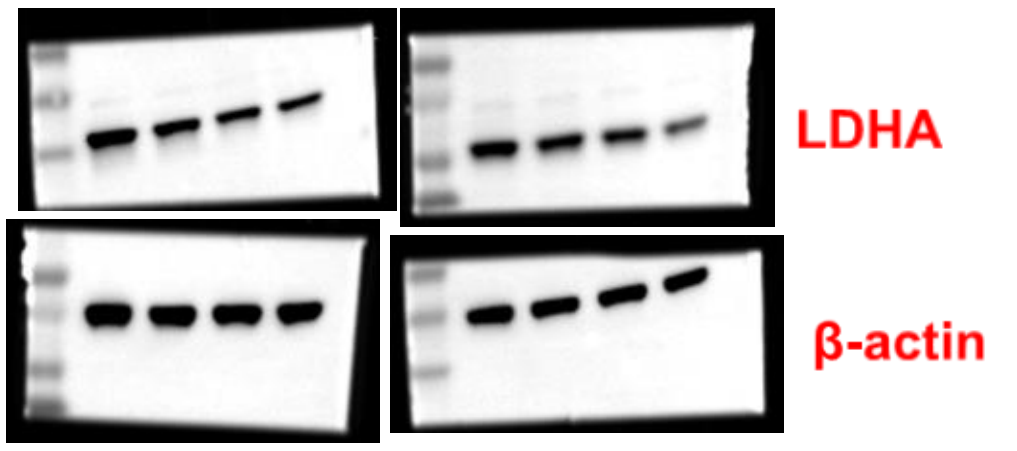

Figure 5A

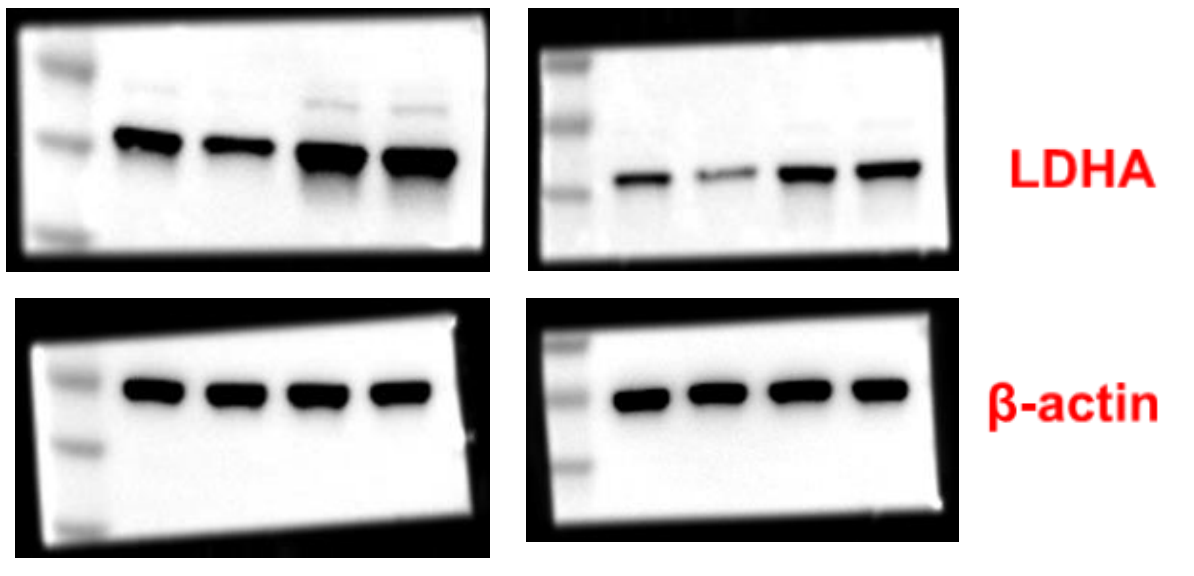

Figure 5C

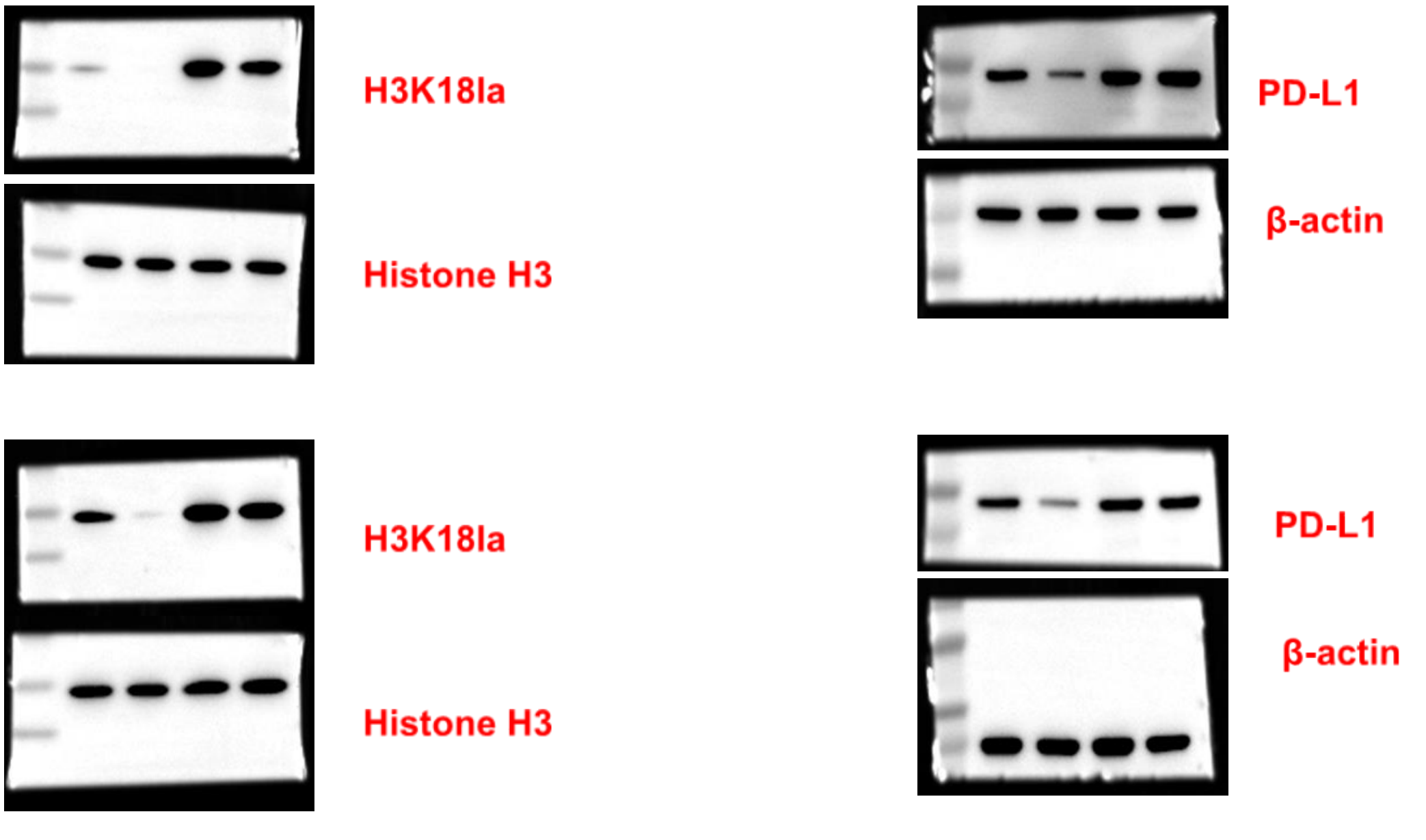

Figure 6A

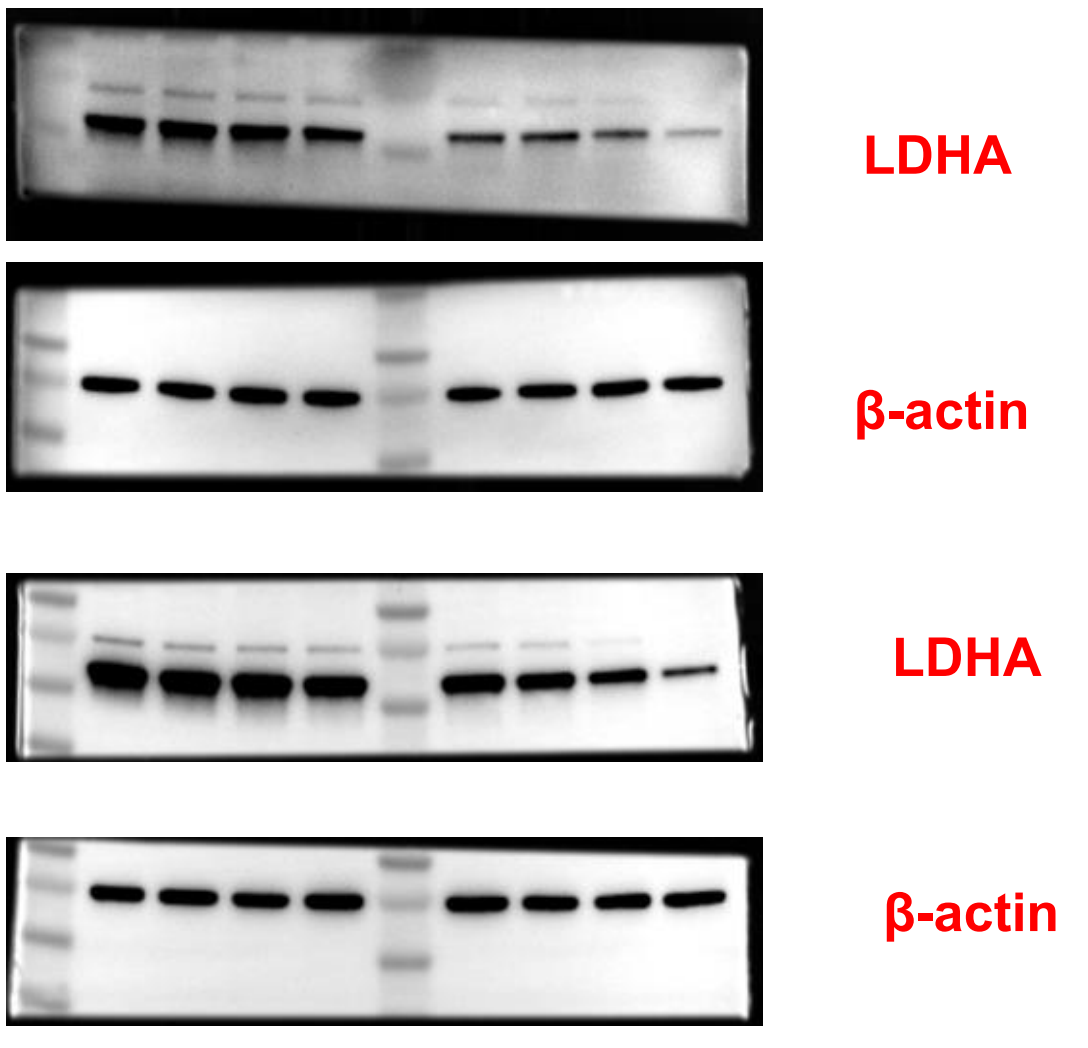

Figure 6C

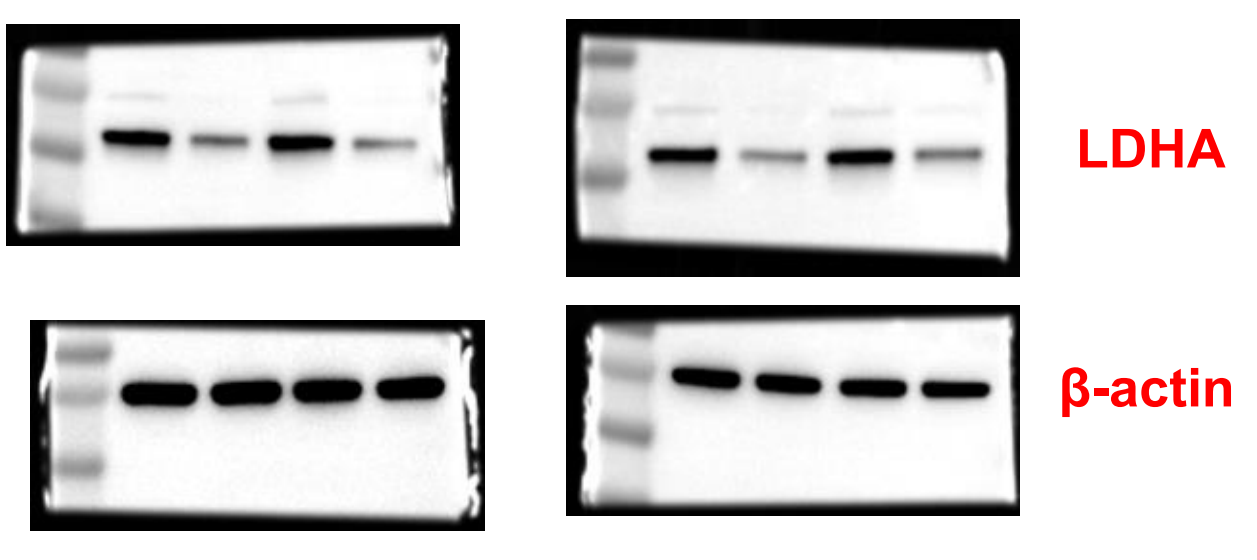

Figure 6D

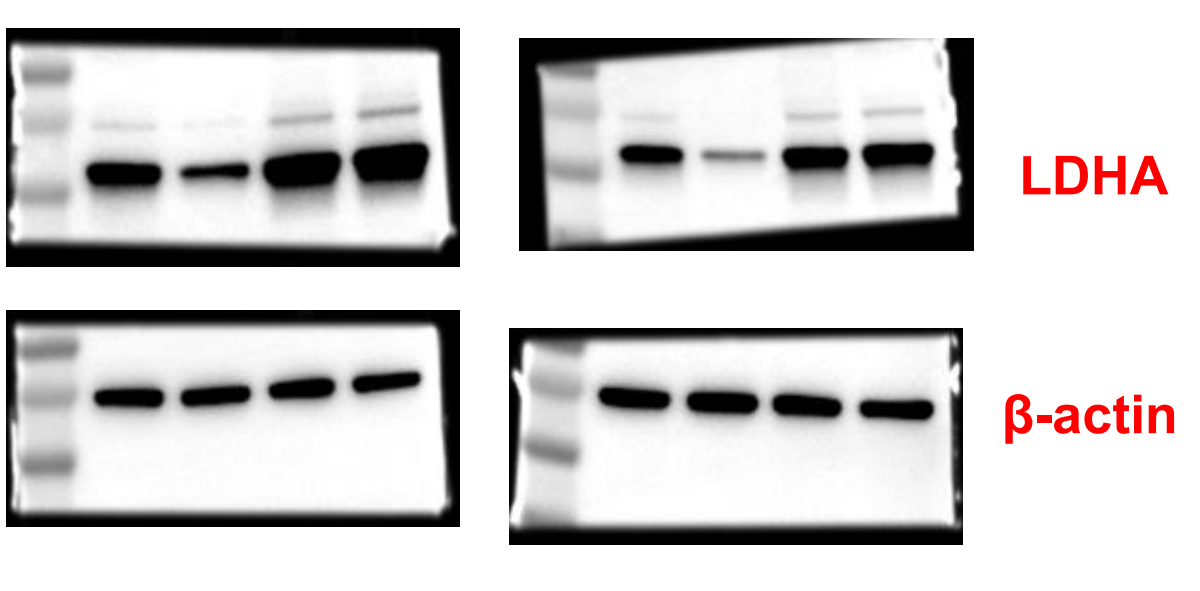

Figure 6E

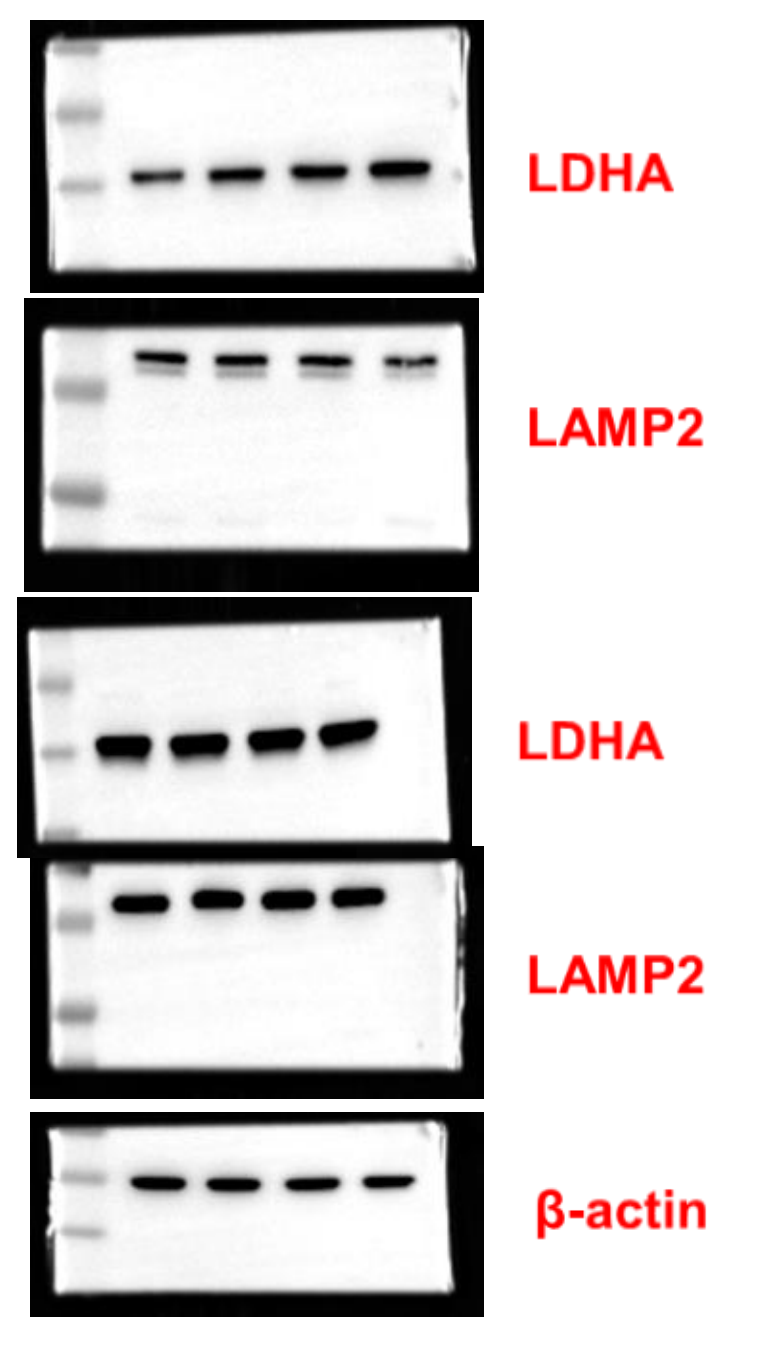

Figure 7A

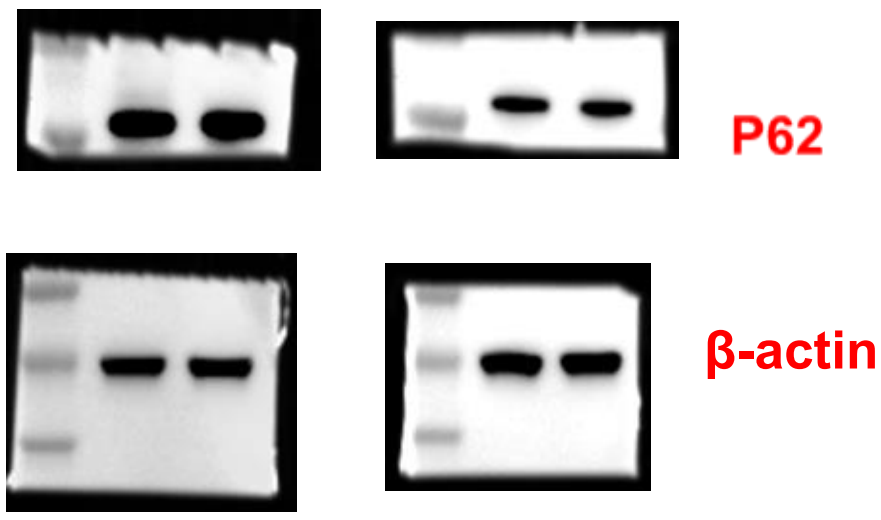

Figure 7B

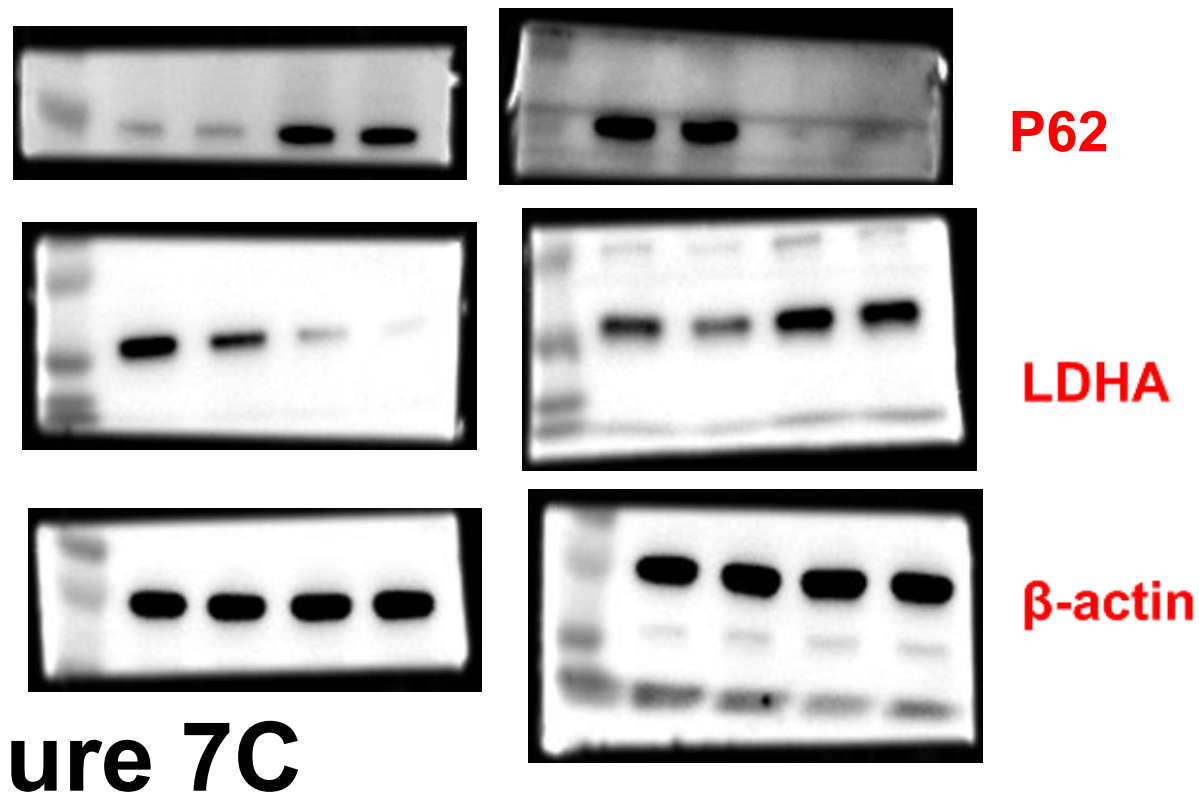

Figure 7C

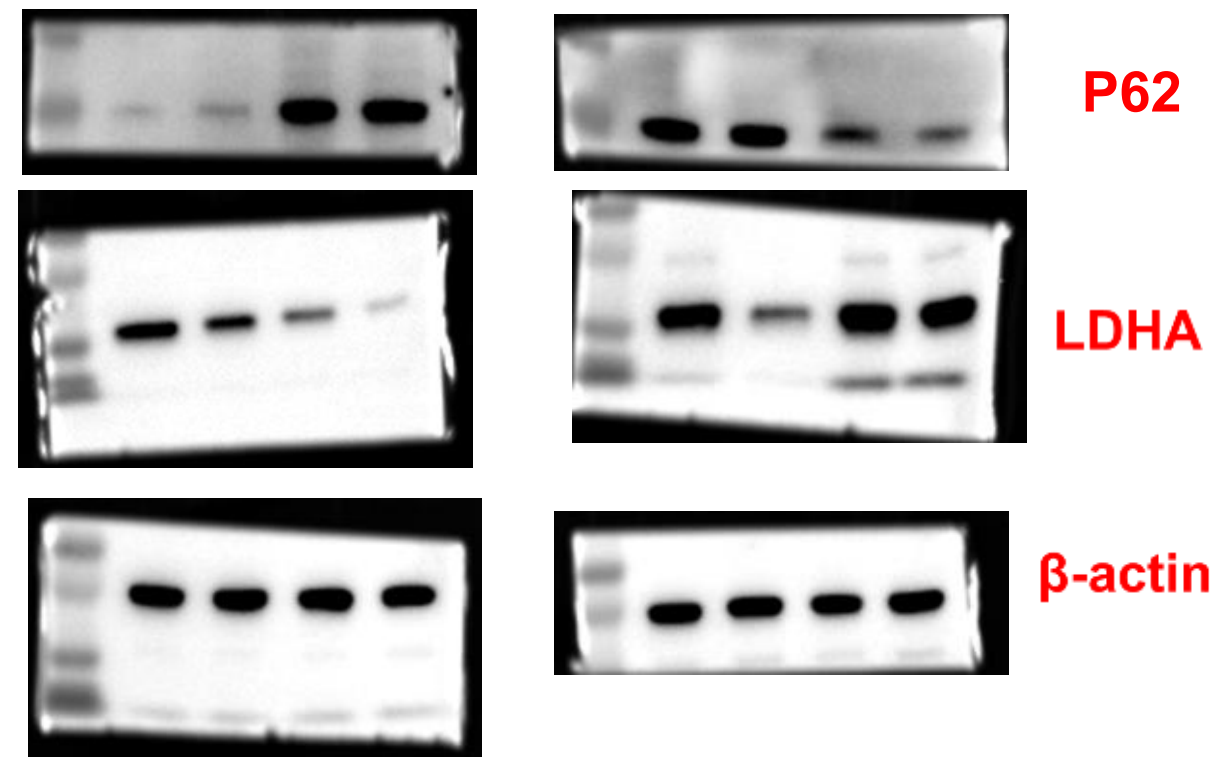

Figure 7D

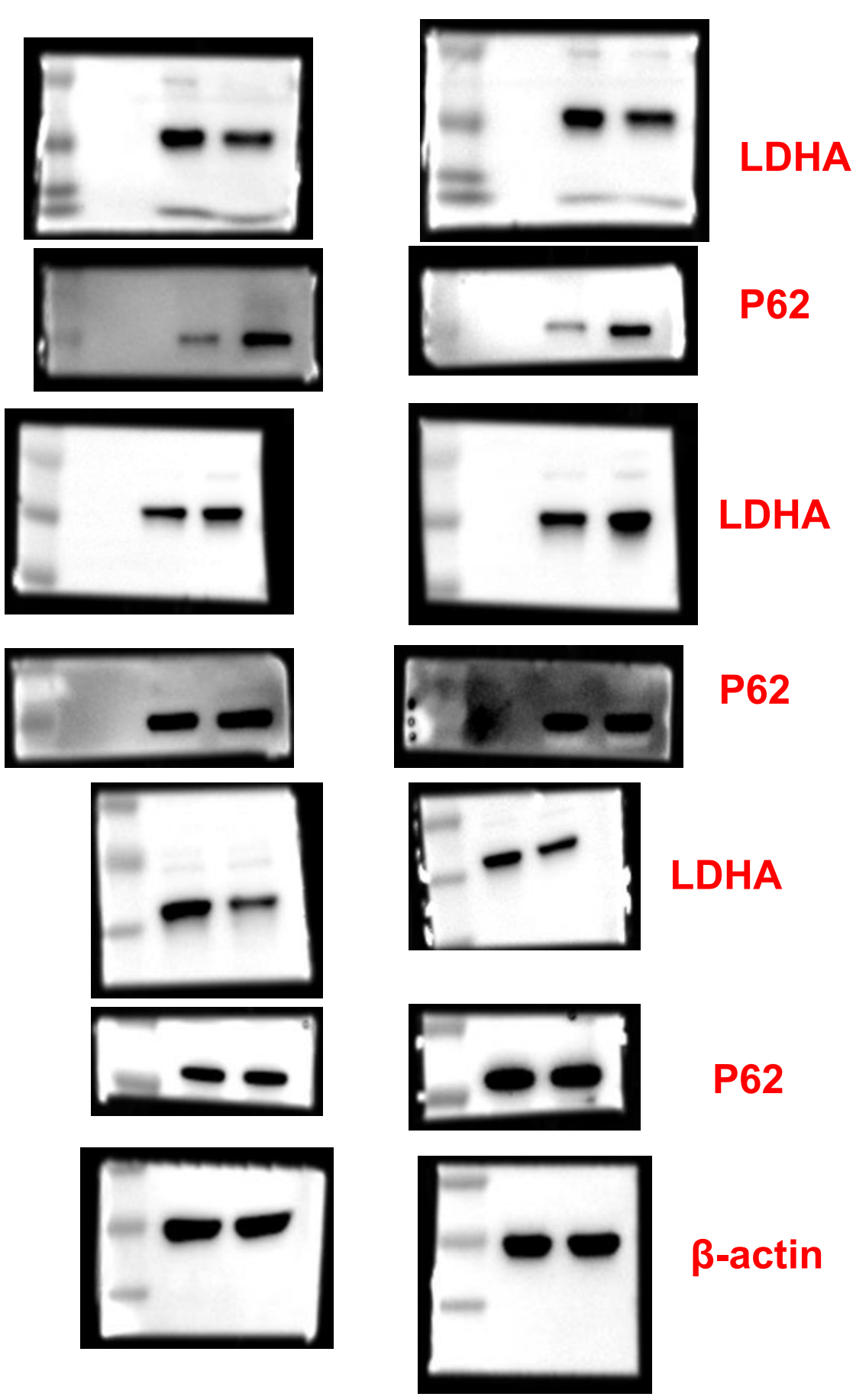

Figure 7E

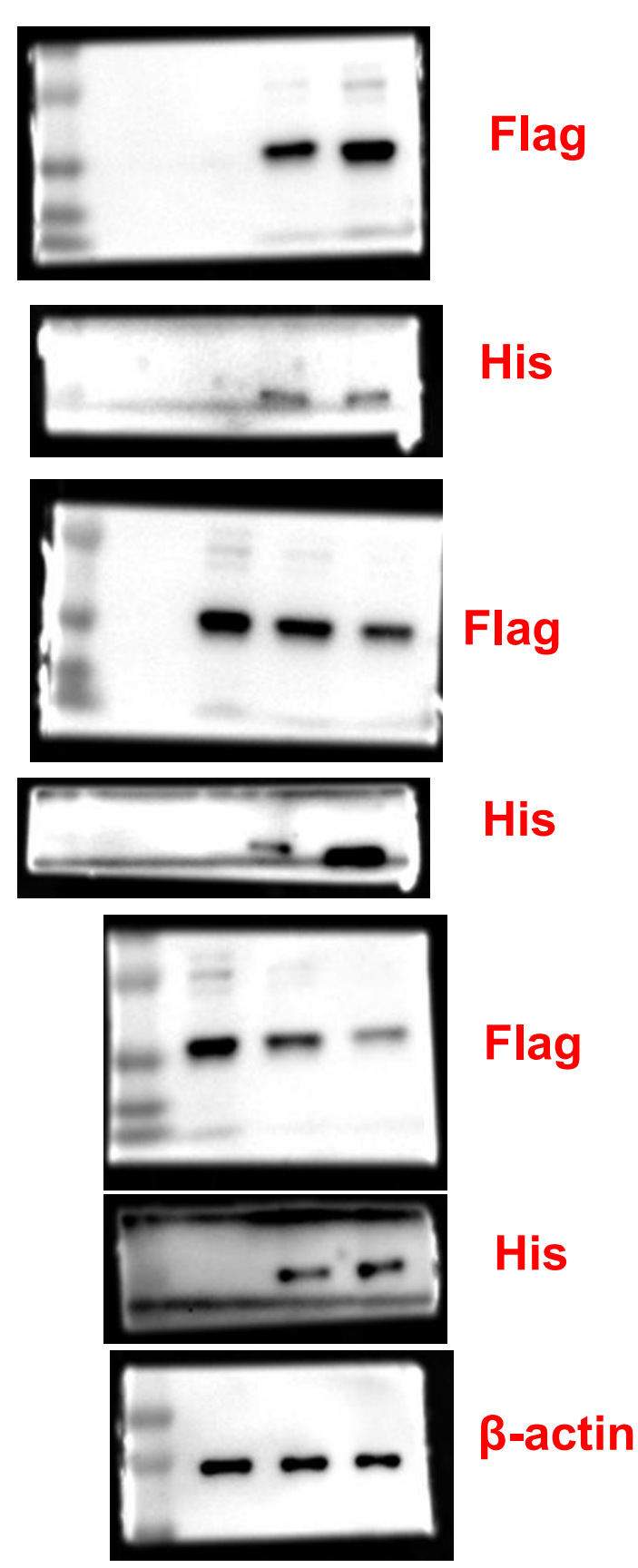

Figure 8C

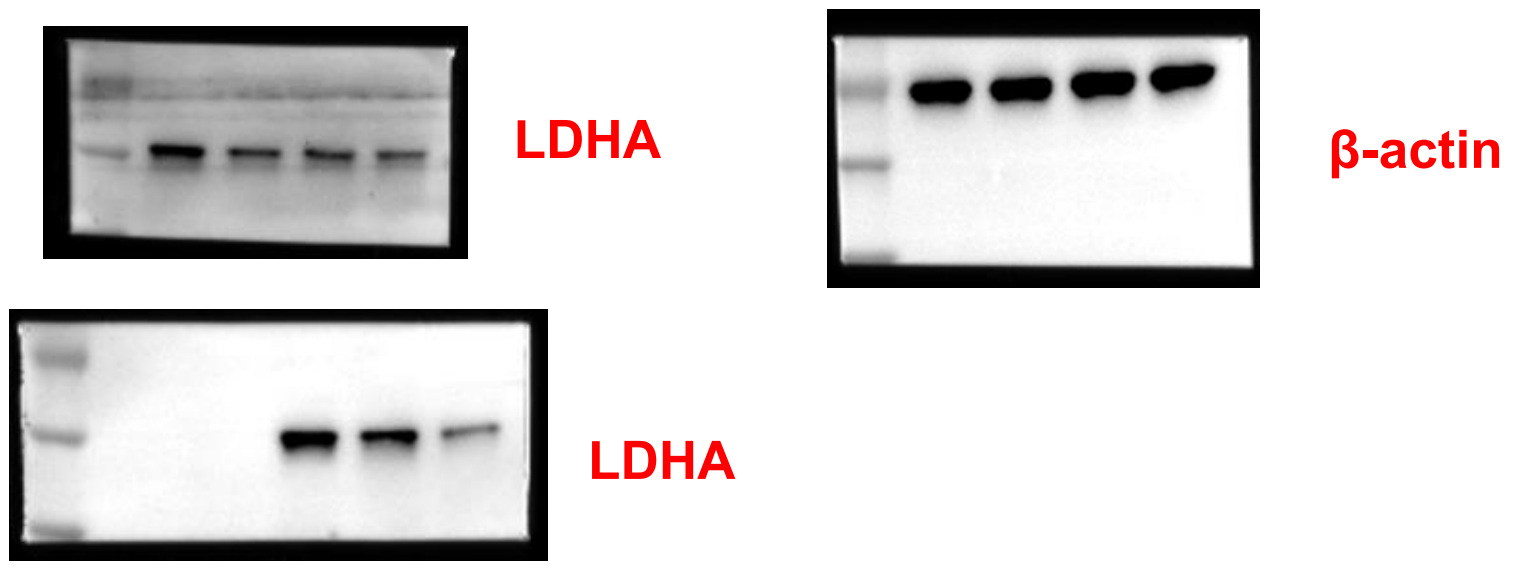

Figure 8D

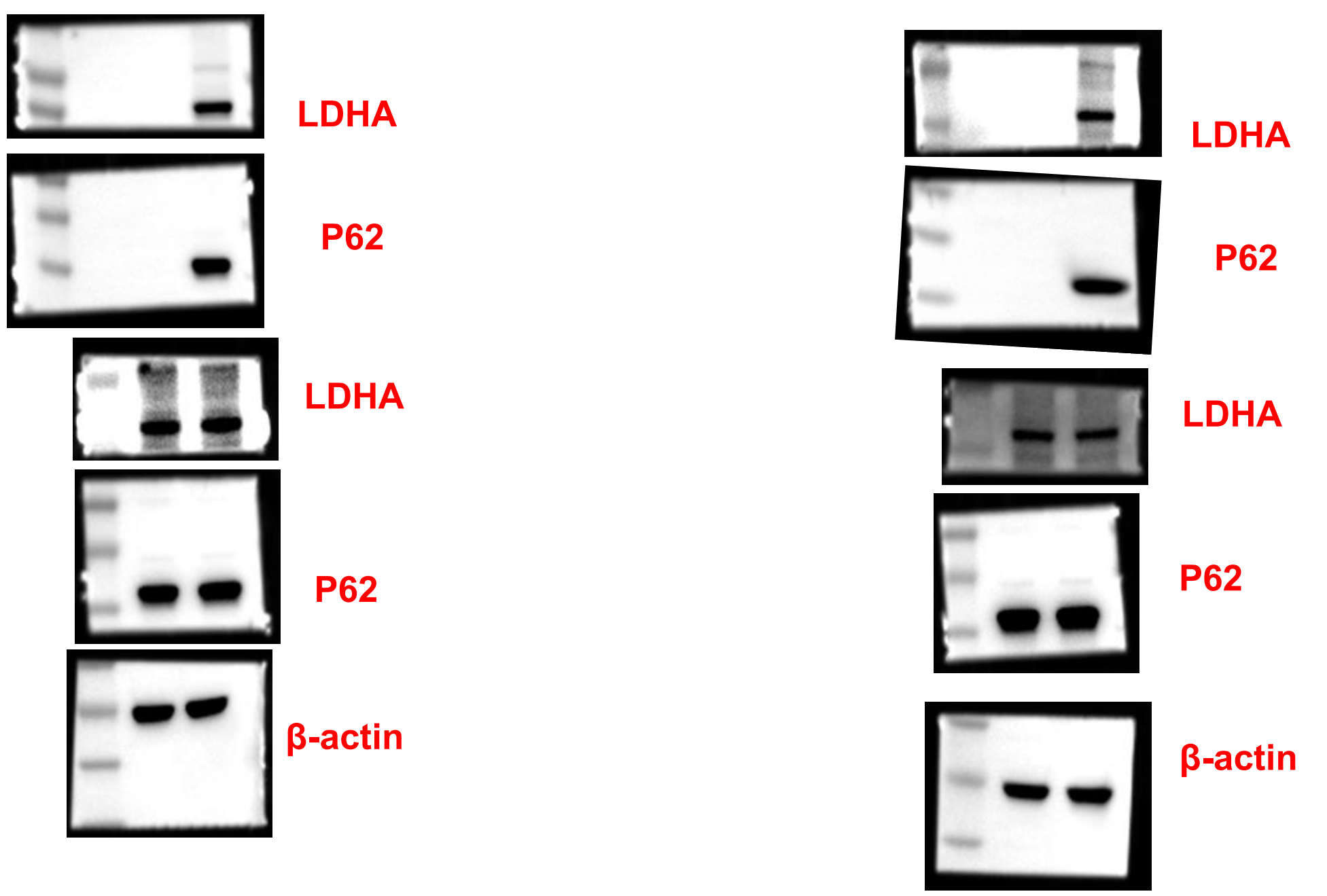

Figure 8F

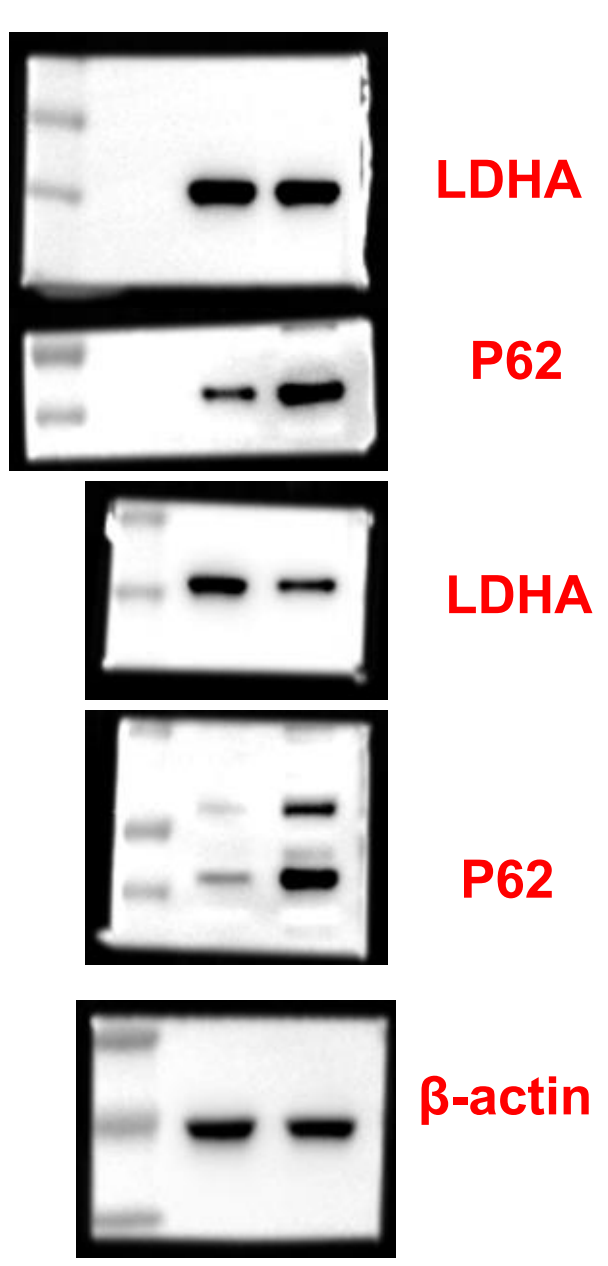

Figure 8G

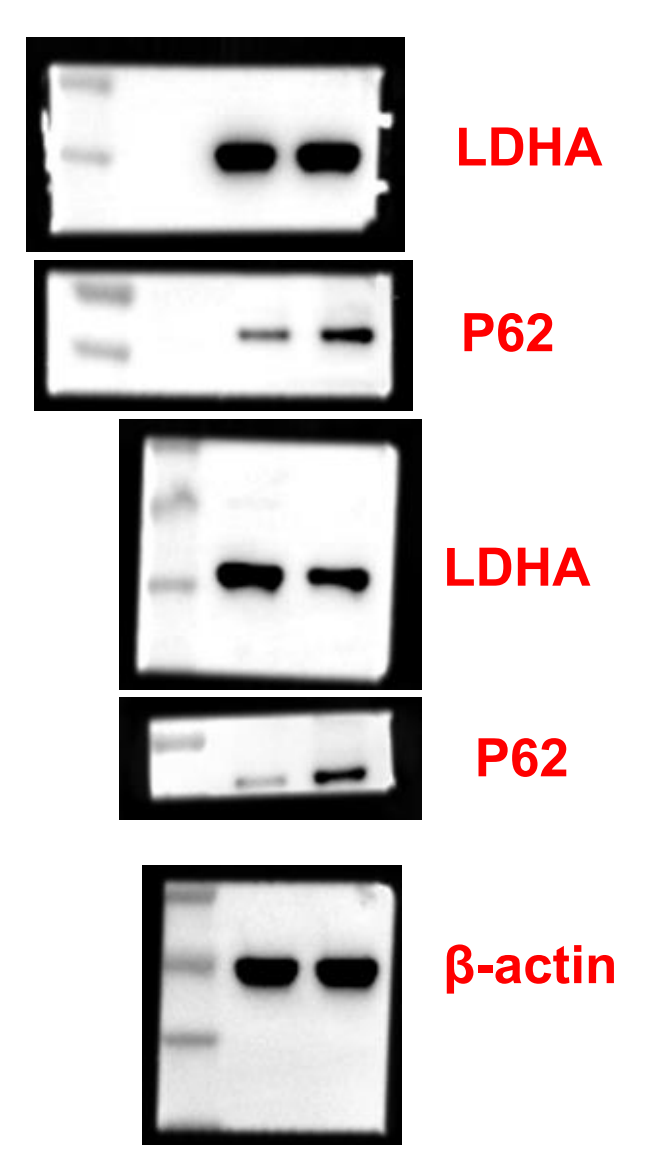

Supplement: Supplementary file 1 [file DataSheet1.pdf]
